# Identification and validation of a T cell receptor targeting KRAS G12V in HLA-A*11:01 pancreatic cancer patients

**DOI:** 10.1172/jci.insight.181873

**Published:** 2025-01-23

**Authors:** Xiongfei Xu, Shiwei Guo, Haihui Gu, Zhanshan Cha, Xiaohan Shi, Xiaoyi Yin, Huan Wang, Suizhi Gao, Bo Li, Lingyu Zhu, Wei Jing, Kailian Zheng, Zhuo Shao, Peng Cheng, Chunhong Zheng, Yi-Ping Shih, Yunguang Li, Baohua Qian, Dong Gao, Eric Tran, Gang Jin

**Affiliations:** 1 Department of Hepatobiliary Pancreatic Surgery,; 2 Shanghai Institute of Pancreatic Diseases, and; 3 Department of Transfusion Medicine, Changhai Hospital, Naval Medical University, Shanghai, China.; 4 Earle A. Chiles Research Institute, Providence Cancer Institute, Portland, Oregon, USA.; 5 International Cancer Institute, Peking University, Beijing, China.; 6 State Key Laboratory of Cell Biology, Shanghai Key Laboratory of Molecular Andrology, Shanghai Institute of Biochemistry and Cell Biology, Center for Excellence in Molecular Cell Science, Chinese Academy of Sciences, Shanghai, China.

**Keywords:** Immunology, Oncology, Cancer immunotherapy, T cell receptor, T cells

## Abstract

T cells targeting a KRAS mutation can induce durable tumor regression in some patients with metastatic epithelial cancer. It is unknown whether T cells targeting mutant KRAS that are capable of killing tumor cells can be identified from peripheral blood of patients with pancreatic cancer. We developed an in vitro stimulation approach and identified HLA-A*11:01–restricted KRAS G12V–reactive CD8^+^ T cells and HLA-DRB1*15:01–restricted KRAS G12V–reactive CD4^+^ T cells from peripheral blood of 2 out of 6 HLA-A*11:01–positive patients with pancreatic cancer whose tumors expressed KRAS G12V. The HLA-A*11:01–restricted KRAS G12V–reactive T cell receptor (TCR) was isolated and validated to specifically recognize the KRAS G12V_8–16_ neoepitope. While T cells engineered to express this TCR specifically recognized all 5 tested human HLA-A*11:01^+^ and KRAS G12V^+^ pancreatic cancer organoids, the recognition was often modest, and tumor cell killing was observed in only 2 out of 5 organoids. IFN-γ priming of the organoids enhanced the recognition and killing by the TCR-engineered T cells. The TCR-engineered T cells could significantly slow the growth of an established organoid-derived xenograft in immunodeficient mice. Our data suggest that this TCR has potential for use in TCR-gene therapy, but additional strategies that enhance tumor recognition by the TCR-engineered T cells likely will be required to increase clinical activity.

## Abstract 2

Screening mutant KRAS-reactive T cells from the peripheral blood of pancreatic cancer patients represents a convenient and effective method to identify mutant KRAS-reactive TCRs.

## Graphical abstract

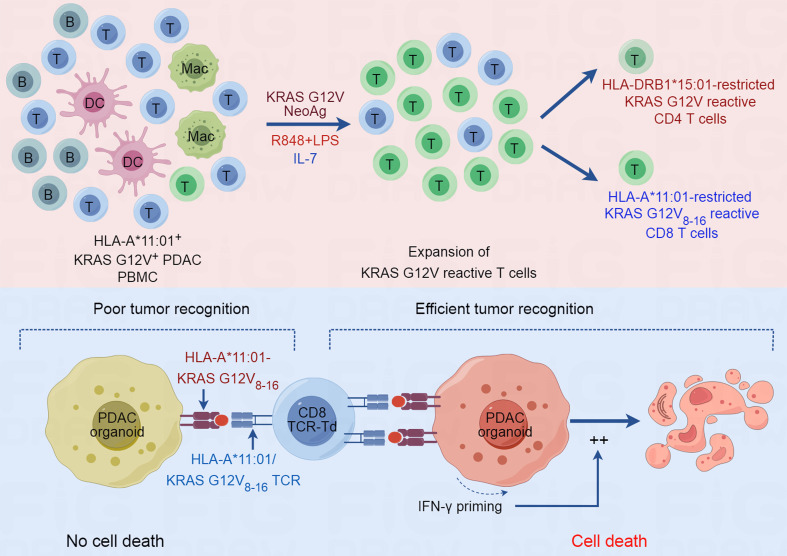

## Introduction

Adoptive cell transfer using tumor-infiltrating lymphocytes (TILs) or T cell receptor–engineered (TCR-engineered) T cells targeting a common oncogenic KRAS mutation can induce durable tumor regression in patients with metastatic epithelial cancer (1, 2), providing a promising strategy for treating solid cancers with KRAS mutations. There are several reasons why KRAS mutations are ideal therapeutic targets for cancer immunotherapy. First, KRAS mutations are found in approximately 20% of all patients with cancer, up to 94% of pancreatic cancers, and up to 45% of colon cancers (3, 4). Importantly, almost all mutations in KRAS occur at either codon 12 or 61, with conserved and recurrent amino acid substitutions, and thus KRAS mutations are shared among patients. Second, some KRAS mutations are immunogenic and can elicit endogenous anti-KRAS T cell responses (5–8), and T cells targeting tumor neoantigens appear to be the main mediators of many effective cancer immunotherapies in humans (9). Third, KRAS mutations are driver “hotspot” mutations that play a critical role in tumorigenesis and are likely to be clonally expressed in all tumor cells present in a patient with cancer (4, 10), and thus targeting KRAS mutations may overcome tumor antigen heterogeneity (8). Therefore, the identification of TCRs targeting mutant KRAS will facilitate the development of TCR-based therapies against mutant KRAS cancers. However, there is a limited number of validated TCRs targeting KRAS mutations; for example, TCRs targeting the following human leukocyte antigen (HLA)/mutant KRAS combinations have been reported: HLA-C*08:02/KRAS G12D (1, 5, 11), HLA-A*11:01/KRAS G12D (7, 12, 13), HLA-A*11:01/KRAS G12V (6–8, 12), HLA-A*03:01/KRAS G12V (6, 7), and HLA-C*01:02/KRAS G12V (13), and some HLA-II–restricted mutant KRAS–reactive TCRs also have been found (13–15). Identifying more functionally avid TCRs targeting KRAS mutations will expand the number of patients potentially eligible for TCR-based therapeutics for patients with mutant KRAS–positive cancers.

Most TCRs targeting KRAS mutations have been isolated and characterized from TILs or peripheral blood of patients with KRAS-mutant epithelial cancers, mostly colorectal cancers (1, 5, 8, 11, 13, 15). Also, HLA-A*11:01–restricted KRAS G12D– or G12V–reactive TCRs have been identified from HLA-A*11:01–transgenic mice vaccinated with KRAS-mutant peptides (12, 16). Recently, TCRs targeting KRAS mutations have been induced in vitro from the peripheral blood mononuclear cells (PBMCs) of HLA-matched healthy donors and identified using a multiomics approach (6) or targeted mass spectrometry (7). Considering that more than 90% of patients with pancreatic ductal adenocarcinoma (PDAC) have KRAS mutations, especially KRAS G12D (41%), G12V (34%), and G12R (16%) mutations (3), TILs or peripheral blood lymphocytes from patients with PDAC may represent ideal sources for screening T cells targeting KRAS mutations, given that these patients may have already mounted an endogenous response against their KRAS mutation. Although CD8^+^ T cell infiltration in PDAC is less abundant than that in melanoma (17), we and others have demonstrated that TILs can be expanded from tumor tissues of PDAC patients, while the success rate and yield of TILs from PDAC patients are highly variable (17–21). An alternative to TILs is peripheral blood lymphocytes from PDAC patients, which may be a more consistent source for screening T cells targeting KRAS mutations, but has the challenge that neoantigen-reactive T cells, if present, are at very low frequencies (8).

To validate in vitro antitumor activity of TCR-engineered T cells, most investigators usually use tumor cell lines that express KRAS mutations and the appropriate HLA, either endogenously expressed or genetically overexpressed (1, 6–8, 12–15). Tumor organoids generated from patient tumor tissues or biopsies can conserve the molecular and cellular compositions of the original tumor, which have the tremendous potential in personalized cancer therapy, particularly preclinical drug screening and predicting patient responses to selected treatment regimens (22–26). Furthermore, tumor organoids have been used to investigate tumor development (26), model the tumor immune microenvironment to facilitate personalized immunotherapy testing (27), generate tumor-reactive T cells from peripheral blood lymphocytes (28, 29), and study the mode of action of engineered T cells in patient cancer organoids (30). However, it is rarely reported that tumor organoids naturally expressing KRAS mutations and specific HLA are used to investigate the recognition and killing of tumor target cells by T cells engineered with TCRs targeting KRAS mutations (31).

Here, we developed a sequential in vitro stimulation approach to expand endogenous KRAS neoantigen–reactive T cells from unfractionated PBMCs of patients with PDAC. Among the 6 PDAC patients with KRAS G12V mutation and HLA-A*11:01 expression, we identified HLA-A*11:01–restricted mutant KRAS G12V–reactive CD8^+^ T cells from one patient that recognized the KRAS G12V_8–16_ neoepitope (VVGAVGVGK), which is predicted to be a strong binder according to NetMHC pan4.0 or NetMHC 4.0 (32, 33). In another patient, we found HLA-DRB1*15:01–restricted mutant KRAS G12V–reactive CD4^+^ T cells. The HLA-A*11:01–restricted mutant KRAS G12V–reactive TCR was isolated and validated to recognize the KRAS G12V_8–16_ neoepitope with high specificity. Furthermore, T cells engineered to express this TCR specifically recognized all 5 tested human pancreatic cancer organoids that naturally expressed KRAS G12V and HLA-A*11:01, although the recognition was generally modest. Accordingly, TCR-transduced T cell killing was observed in only 2 out of 5 organoids. However, the recognition of human pancreatic cancer organoids mediated by this TCR could be enhanced by interferon γ (IFN-γ) priming of organoids. Adoptive transfer of the TCR-engineered T cells also could significantly slow the growth of an established organoid xenograft in immunodeficient mice. Our preclinical data demonstrate the therapeutic potential of this TCR and also highlight the likely need for combination with other therapies that can increase tumor recognition by the TCR-engineered T cells in order to increase antitumor efficacy.

## Results

### Screening for KRAS G12V–reactive T cells from PBMCs of HLA-A*11:01–expressing patients with pancreatic cancer.

**Figure 1:**
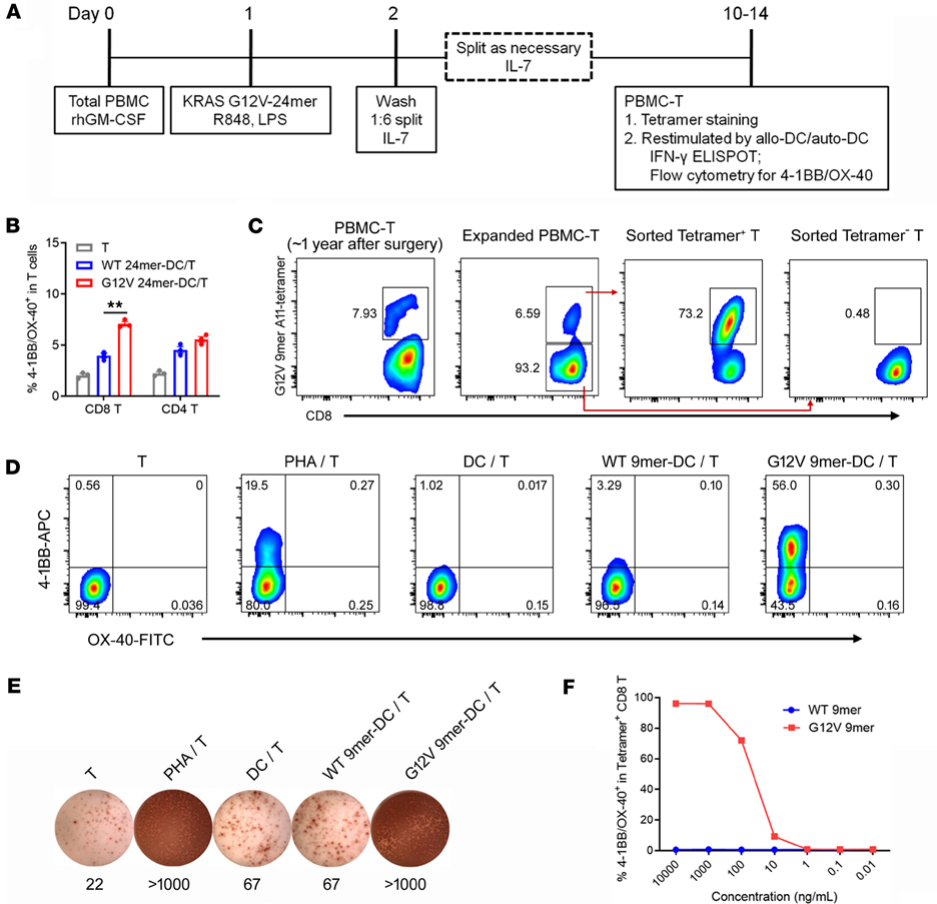
Identification and functional analysis of HLA-A*11:01–restricted mutant KRAS G12V–reactive CD8^+^ T cells. (**A**) Diagram of patient PBMC–derived potential KRAS G12V–reactive T cell (PBMC-T) culture. (**B**) PBMC-Ts from Pt.001 PBMCs from approximately 1 year after surgery were cocultured overnight with allogeneic HLA-A*11:01–expressing DCs (from Pt.007) pulsed with KRAS WT or G12V 24-mer peptides. The reactivity of T cells was tested by 4-1BB/OX-40 upregulation. Data are presented as mean ± SEM from 3 technical replicates. **P < 0.01 by 1-way ANOVA followed by Tukey’s multiple-comparison test. (**C**) Various T cells, including PBMC-T, expanded PBMC-T (PBMC-T expanded with autologous PBMCs), sorted tetramer^+^ CD8^+^ T cells, and sorted tetramer^–^ CD8^+^ T cells (further expanded with irradiated feeder cells) were stained with KRAS G12V 9-mer–HLA-A*11:01 tetramer. The numbers in plots indicate the percentage of tetramer^+^ CD8^+^ T cells among CD8^+^ T cells. (**D** and **E**) Expanded tetramer^+^ CD8^+^ T cells were cocultured overnight with autologous DCs pulsed with 2 μg/mL KRAS WT or G12V 9-mer peptides. 4-1BB/OX-40 upregulation (**D**) and ELISPOT IFN-γ secretion (**E**) were assayed. The numbers in plots indicate the percentage of different subsets among tetramer^+^ CD8^+^ T cells. (**F**) Expanded tetramer^+^ CD8^+^ T cells were cocultured overnight with naturally HLA-A*11:01–expressing Panc-1 cells pulsed with various concentrations of KRAS WT or G12V 9-mer peptides. 4-1BB/OX-40 upregulation was assayed by flow cytometry.

Due to the very low frequency of neoantigen-reactive T cells in peripheral blood (8), we tried to enrich endogenous KRAS neoantigen–reactive T cells from patient unfractionated PBMCs using a modified in vitro stimulation approach (34). As depicted in Figure 1A, unfractionated PBMCs were exposed to GM-CSF plus long peptide plus Toll-like receptor (TLR) agonists (resiquimod [R848] and lipopolysaccharide [LPS]) to enhance antigen presentation and cross-presentation, and then exposed to IL-7 to promote the expansion of endogenous neoantigen-reactive CD8^+^ or CD4^+^ T cells. In preliminary experiments, unfractionated PBMCs from healthy donors were exposed to different TLR agonists, and TLR8 agonist R848 plus TLR4 agonist LPS could significantly induce IL-12 p70 production (Supplemental Figure 1; supplemental material available online with this article; https://doi.org/10.1172/jci.insight.181873DS1), which might contribute to the enhancement of antigen presentation and cross-presentation.

**Figure 2:**
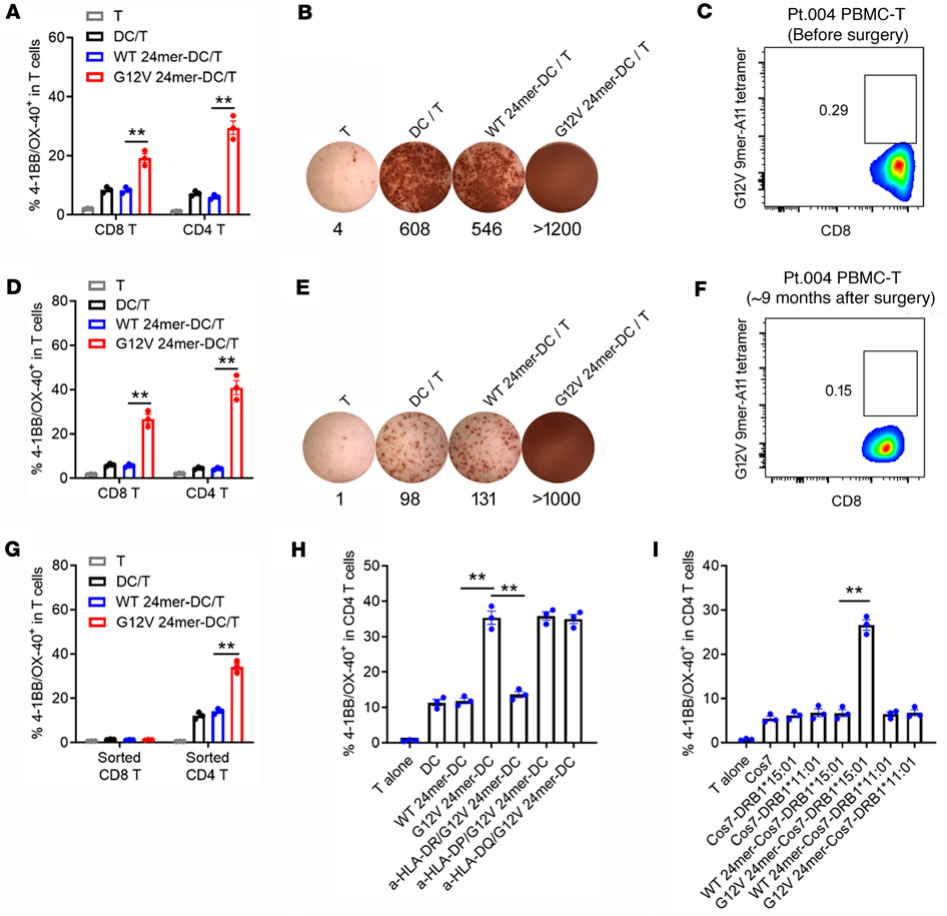
Identification of HLA-DRB1*15:01–restricted mutant KRAS G12V–reactive CD4^+^ T cells. (**A** and **B**) PBMC-Ts (batch 1) from Pt.004 PBMCs before surgery were cocultured overnight with allogeneic HLA-A*11:01–expressing DCs (from Pt.007) pulsed with KRAS WT or G12V 24-mer peptides. The reactivity of T cells was tested by 4-1BB/OX-40 upregulation (**A**) and ELISPOT IFN-γ secretion (**B**) assays. (**C**) PBMC-Ts (batch 1) from Pt.004 were stained with KRAS G12V 9-mer–HLA-A*11:01 tetramer. The numbers in plots indicate the percentage of tetramer^+^ CD8^+^ T cells among CD8^+^ T cells. (**D** and **E**) PBMC-Ts (batch 2) from Pt.004 PBMCs from approximately 9 months after surgery were cocultured overnight with autologous DCs pulsed with KRAS WT or G12V 24-mer peptides. 4-1BB/OX-40 upregulation (**D**) and ELISPOT IFN-γ secretion (**E**) were assayed. (**F**) PBMC-Ts (batch 2) from Pt.004 were stained with KRAS G12V 9-mer–HLA-A*11:01 tetramer. The numbers in the plot indicate the percentage of tetramer^+^ CD8^+^ T cells among CD8^+^ T cells. (**G**) Sorted CD8^+^ or CD4^+^ T cells from Pt.004 PBMC-Ts (batch 2) were cocultured overnight with autologous DCs pulsed with KRAS WT or G12V 24-mer peptides. 4-1BB/OX-40 upregulation was assayed. (**H**) Sorted CD4^+^ T cells from Pt.004 PBMC-Ts (batch 2) were cocultured overnight with autologous DCs pulsed with KRAS WT or G12V 24-mer peptides in the presence of various anti–HLA-DR, –HLA-DP, or –HLA-DQ antibodies. 4-1BB/OX-40 upregulation was assayed. (**I**) Sorted CD4^+^ T cells from Pt.004 PBMC-Ts (batch 2) were cocultured overnight with Cos7 cells transiently transfected with plasmids encoding HLA-DRB1*15:01 or HLA-DRB1*11:01 and then pulsed with KRAS WT or G12V 24-mer peptides. 4-1BB/OX-40 upregulation was assayed. Data are presented as mean ± SEM from 3 technical replicates. **P < 0.01 by 1-way ANOVA followed by Tukey’s multiple-comparison test (**A**, **D**, and **G**–**I**).

To screen mutant KRAS–reactive T cells from PBMCs of human pancreatic cancer patients more precisely, we used the peptide/MHC prediction algorithms NetMHC pan4.0 and NetMHC 4.0 (32, 33) to predict potential immunogenic KRAS neoantigens and found that the HLA-A*11:01–restricted KRAS G12V 9-mer (VVGAVGVGK) was predicted to bind strongly to HLA-A*11:01 (Supplemental Tables 1 and 2). We focused on human pancreatic cancer patients with KRAS G12V mutation and HLA-A*11:01 expression detected by whole-exome sequencing. In our prospective pancreatic cancer biobank, from July 2019 to April 2020, there were 6 PDAC patients with KRAS G12V mutation and HLA-A*11:01 expression who had frozen PBMCs from approximately 10 mL of peripheral blood before or after surgery. We stimulated PBMCs for 10–14 days with KRAS G12V peptides in an attempt to enrich KRAS G12V–reactive T cells (PBMC-Ts) from these 6 patients (Figure 1A). We tested whether there were KRAS G12V–reactive CD8^+^ or CD4^+^ T cells using tetramer staining or T cell activation after restimulation by antigen-pulsed allogeneic or autologous dendritic cells (DCs). We identified HLA-A*11:01–restricted mutant KRAS G12V–reactive CD8^+^ T cells from PBMC-Ts of one patient (Pt.001) (Figure 1) and mutant KRAS G12V–reactive CD4^+^ T cells from PBMC-Ts of another patient (Pt.004) (Figure 2), while we did not detect KRAS G12V–reactive T cells from PBMC-Ts of the other 4 patients (Pt.002, Pt.003, Pt.005, and Pt.006; Supplemental Figure 2).

### Characterization of HLA-A*11:01–restricted mutant KRAS G12V–reactive CD8^+^ T cells.

To identify T cells targeting mutant KRAS G12V, cultured PBMC-Ts from Pt.001 PBMCs from approximately 1 year after surgery were cocultured with HLA-A*11:01^+^ allogeneic DCs pulsed with KRAS WT or G12V 24-mer long peptides. As shown in Figure 1B, PBMC-Ts from Pt.001 showed more reactivity against KRAS G12V 24-mer long peptide than that of KRAS WT, indicating the possible existence of mutant KRAS G12V–reactive T cells. Indeed, KRAS G12V 9-mer–HLA-A*11:01 tetramer staining demonstrated that there were 7.93% tetramer^+^ CD8^+^ T cells among total CD8^+^ T cells in PBMC-Ts from Pt.001 (Figure 1C). These PBMC-Ts were further expanded with KRAS G12V 24-mer long peptide–pulsed autologous PBMCs for approximately 10 days, with little change in the percentage of tetramer^+^ CD8^+^ T cells after this expansion (Figure 1C). Next, we sorted tetramer^+^ CD8^+^ T cells, expanded them using an anti-CD3 antibody and irradiated feeder cells, and then detected tetramer staining and tested the reactivity of the cells against mutant KRAS G12V. As shown in Figure 1C, 73.2% of these expanded tetramer^+^ CD8^+^ T cells bound KRAS G12V 9-mer–HLA-A*11:01 tetramer. Importantly, these expanded tetramer^+^ CD8^+^ T cells showed selective and strong reactivity against autologous antigen-presenting cells pulsed with KRAS G12V 9-mer, but not KRAS WT 9-mer, by 4-1BB/OX-40 upregulation and ELISPOT IFN-γ secretion assays (Figure 1, D and E). To evaluate the potency of these expanded tetramer^+^ CD8^+^ T cells, we cocultured them with naturally HLA-A*11:01–expressing Panc-1 cells pulsed with a serial dilution of KRAS WT or G12V 9-mer peptides (Figure 1F). The results showed that these expanded tetramer^+^ CD8^+^ T cells selectively recognize the KRAS G12V mutation presented on HLA-A*11:01 at concentrations as low as 10 ng/mL based on 4-1BB/OX-40 upregulation (Figure 1F). Notably, we did not detect KRAS G12V 9-mer–HLA-A*11:01 tetramer^+^ CD8^+^ T cells in cultured PBMC-Ts from 2 other batches of PBMCs, including PBMCs from about 1 year after surgery and about 2 years after surgery (Supplemental Figure 3), indicating that mutant KRAS G12V–reactive CD8^+^ T cells could not always be expanded from PBMCs of this patient at different postoperation times, potentially due to a low frequency of the reactive T cells in blood.

### Identification of HLA-DRB1*15:01–restricted mutant KRAS G12V–reactive CD4^+^ T cells.

In another patient (Pt.004), we identified mutant KRAS G12V–reactive CD4^+^ T cells in cultured PBMC-Ts from PBMCs before surgery (Figure 2, A–C) and PBMCs from approximately 9 months after surgery (Figure 2, D–G). As shown in Figure 2, A and B, cultured PBMC-Ts from PBMCs before surgery showed more reactivity against KRAS G12V 24-mer long peptide–pulsed allogeneic DCs than that of KRAS WT. Also, cultured PBMC-Ts from PBMCs from approximately 9 months after surgery showed more reactivity against KRAS G12V 24-mer long peptide–pulsed autologous DCs than that of KRAS WT (Figure 2, D and E). These results indicated that there might be mutant KRAS G12V–reactive CD8^+^ T or CD4^+^ T cells in these 2 batches of PBMC-Ts. However, KRAS G12V 9-mer–HLA-A*11:01 tetramer staining showed that the percentages of tetramer^+^ CD8^+^ T among total CD8^+^ T cells of these 2 batches of PBMC-Ts were low (<1%; Figure 2, C and F), indicating that the reactivity of CD8^+^ T cells was possibly due to nonspecific activation. Indeed, sorted CD4^+^ T cells, but not sorted CD8^+^ T cells, from batch 2 PBMC-Ts showed more reactivity against KRAS G12V 24-mer long peptide–pulsed autologous DCs than that of KRAS WT (Figure 2G). Considering that there were several shared HLA-II alleles between allogeneic Pt.007 DCs and autologous Pt.004 DCs (Supplemental Table 3), HLA restriction of mutant KRAS G12V–reactive CD4^+^ T cells could be one of these shared HLA-II alleles. Using HLA-II–blocking antibodies, it was demonstrated that mutant KRAS G12V–reactive CD4^+^ T cells were HLA-DR dependent (Figure 2H). Furthermore, HLA-DRB1*15:01–, but not HLA-DRB1*11:01–transfected, Cos7 cells could present mutant KRAS G12V 24-mer to activate sorted CD4^+^ T cells from Pt.004 PBMC-Ts (Figure 2I and Supplemental Figure 4), indicating that mutant KRAS G12V–reactive CD4^+^ T cells were HLA-DRB1*15:01 restricted.

### Identification of HLA-A*11:01–restricted TCR targeting mutant KRAS G12V and establishment of TCR-transduced T cells.

**Figure 3:**
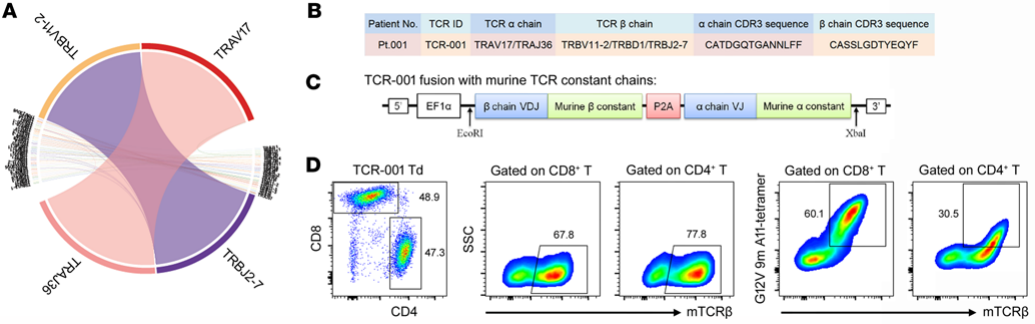
Identification of HLA-A*11:01–restricted TCR targeting mutant KRAS G12V and establishment of TCR-transduced T cells. (**A**) KRAS G12V 9-mer–HLA-A*11:01 tetramer^+^ CD8^+^ T cells from Pt.001 were sorted for bulk transcriptomic TCR sequencing. The frequencies of all the clonotypes with given V-J junctions are shown. (**B**) TCR information and CDR3 sequences of the major clonotype (TCR-001) from KRAS G12V 9-mer–HLA-A*11:01 tetramer^+^ CD8 T cells of Pt.001. (**C**) Diagram of TCR-001 variable chain fusions with murine TCR constant chains. (**D**) Transduction efficiency and tetramer staining of lentivirally transduced allogeneic healthy donor pan T cells expressing TCR-001 assayed by flow cytometry. The numbers in plots indicate the percentage of corresponding cell subsets among gated T cells.

To isolate the HLA-A*11:01–restricted mutant KRAS G12V–reactive TCR(s) from Pt.001, sorted high-purity tetramer^+^ CD8^+^ T cells from expanded tetramer^+^ CD8^+^ T cells were used for bulk transcriptomic TCR sequencing, which identified 1 major TCR clonotype (TCR-001) (Figure 3, A and B). To further test this TCR, we synthesized, cloned, and lentivirally transduced the TCR into allogeneic healthy donor pan T cells (Figure 3C and Supplemental Figure 5). Murine TCR α and β constant regions were used in place of the human constant regions to promote pairing of the introduced TCR (21, 35). As shown in Figure 3D, transduction efficiency of TCR-001–transduced CD8^+^ and CD4^+^ T cells was greater than 65% and the percentage of tetramer^+^ CD8^+^ T cells was greater than 60%.

### TCR-001–transduced T cells specifically recognized endogenously processed and presented antigen and human pancreatic cancer cell lines with KRAS G12V mutation and HLA-A*11:01 expression.

**Figure 4:**
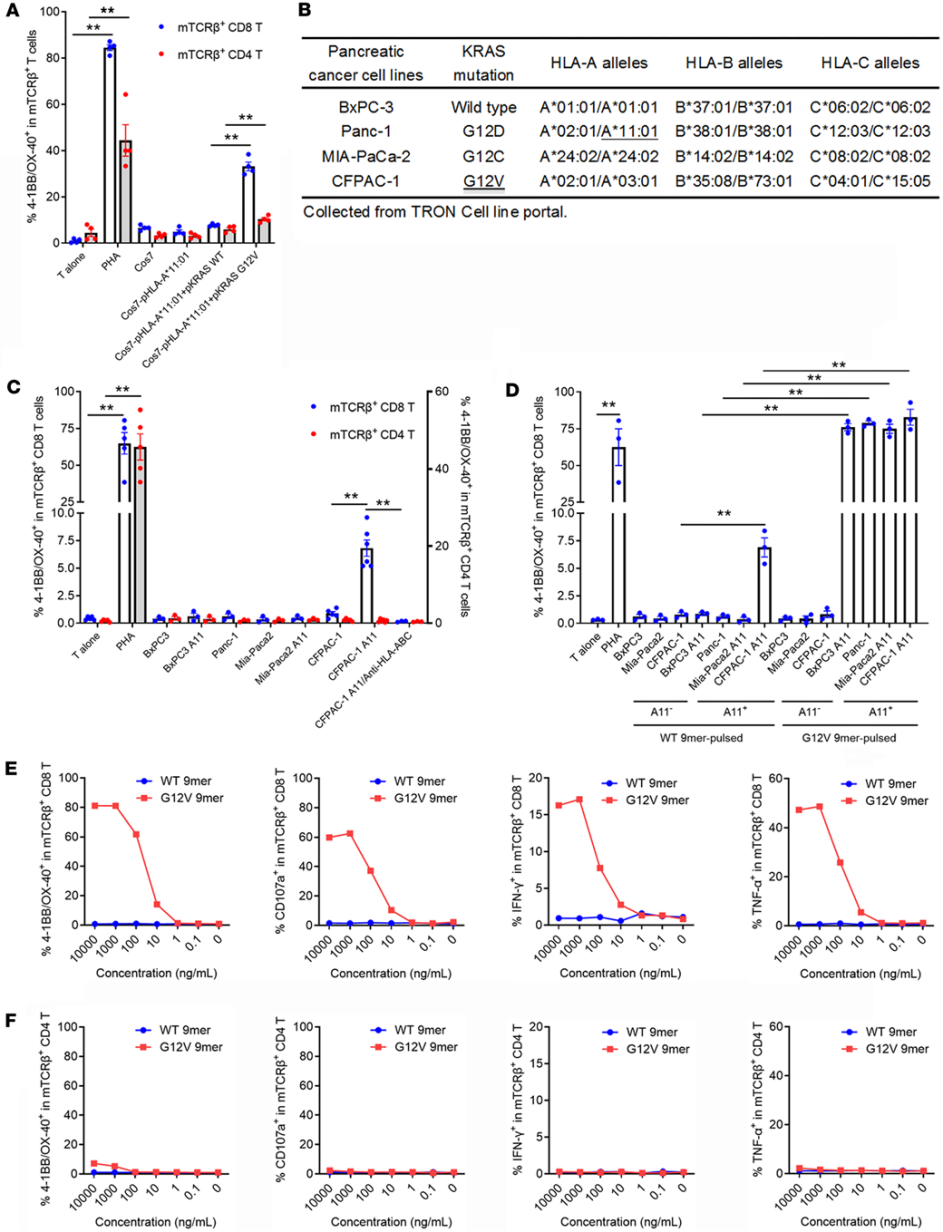
TCR-001–transduced T cells specifically recognized endogenously processed and presented antigen and human pancreatic cancer cell lines. (**A**) TCR-001–transduced allogeneic T cells were cocultured overnight with Cos7 cells transiently transfected with plasmids encoding HLA-A*11:01 and the KRAS G12V or WT full-length gene. 4-1BB/OX-40 upregulation was assayed by flow cytometry. Error bars represent SEM of 4 biological replicates. Dots indicate biological replicates. **P < 0.01. (**B**) KRAS mutation and HLA profiles of human pancreatic cancer cell lines. Information is from the TRON Cell Line portal (https://github.com/TRON-Bioinformatics/TCLP). (**C**) TCR-001–transduced allogeneic T cells were cocultured overnight with human pancreatic cancer cell lines naturally expressing KRAS G12 mutations with and without HLA-A*11:01 transfection. 4-1BB/OX-40 upregulation was assayed by flow cytometry. Anti–human HLA-ABC antibody (20 μg/mL) was used in 1 group. Error bars represent SEM of at least 3 biological replicates. Dots indicate biological replicates. **P < 0.01. (**D**) TCR-001–transduced allogeneic T cells were cocultured overnight with human pancreatic cancer cell lines with and without HLA-A*11:01 transfection pulsed with 1 μg/mL KRAS WT or G12V 9-mer peptides. 4-1BB/OX-40 upregulation was assayed by flow cytometry. Error bars represent SEM of at least 3 biological replicates. Dots indicate biological replicates. **P < 0.01. (**E** and **F**) TCR-001–transduced allogeneic T cells were cocultured with naturally HLA-A*11:01–expressing Panc-1 cells pulsed with various concentrations of KRAS WT or G12V 9-mer peptides overnight or for 5 hours. 4-1BB/OX-40 upregulation was assayed by flow cytometry. CD107a upregulation and IFN-γ/TNF-α secretion were assayed by intracellular cytokine staining. The results of mTCRβ^+^ CD8^+^ T cells (**D**) and mTCRβ^+^ CD4^+^ T cells (**E**) are shown. Statistical differences were determined with 1-way ANOVA followed by Tukey’s multiple-comparison test (**A** and **D**) or mixed-effects analysis with Tukey’s multiple-comparison test (**C**).

To evaluate specific recognition of TCR-001 against endogenously processed and presented antigen, we cocultured TCR-001–transduced allogeneic T cells with Cos7 cells transiently transfected with HLA-A*11:01 (Supplemental Figure 6) and KRAS G12V or the WT full-length gene (Supplemental Figure 7). The results showed that TCR-001–transduced CD8^+^ T cells selectively and significantly recognized Cos7 cells expressing HLA-A*11:01 and the KRAS G12V full-length gene, but not the KRAS WT full-length gene, while weak effects of TCR-001–transduced CD4^+^ T cells against Cos7 cells expressing HLA-A*11:01 and the KRAS G12V full-length gene were observed (Figure 4A), indicating that TCR-001 specifically recognized endogenously processed and presented antigen.

To further evaluate the specificity of TCR-001, we cocultured TCR-001–transduced allogeneic T cells with human pancreatic cancer cell lines naturally expressing KRAS G12 mutations (Figure 4B) with or without HLA-A*11:01 transfection (Supplemental Figure 6). The results showed that TCR-001–transduced CD8^+^ T cells, but not CD4^+^ T cells, selectively recognized CFPAC-1 A11 cells naturally expressing the KRAS G12V mutation, and this reactivity was abrogated by blocking HLA-I (Figure 4C). Furthermore, TCR-001–transduced CD8^+^ T cells selectively recognized mutant KRAS G12V 9-mer peptide–pulsed pancreatic cancer cell lines that were transfected to express HLA-A*11:01 (Figure 4D). These results demonstrated that TCR-001 specifically targeted the KRAS G12V mutation with HLA-A*11:01 restriction.

To evaluate the potency of TCR-001, we cocultured TCR-001–transduced allogeneic T cells with naturally HLA-A*11:01–expressing Panc-1 cells pulsed with a serial dilution of KRAS WT or G12V 9-mer peptides (Figure 4, E and F). The results of 4-1BB/OX-40 upregulation, CD107a upregulation, and IFN-γ/tumor necrosis factor α (TNF-α) secretion showed that TCR-001–transduced allogeneic CD8^+^ T cells, but not CD4^+^ T cells, selectively recognized the KRAS G12V mutation presented on HLA-A*11:01 at concentrations as low as 10 ng/mL (Figure 4, E and F), similar to the minimum concentration recognized by the above expanded tetramer^+^ CD8^+^ T cells from which the TCR was derived (Figure 1F).

### TCR-001–transduced T cells specifically recognized all 5 tested human pancreatic cancer organoids, but only killed some of them.

**Figure 5:**
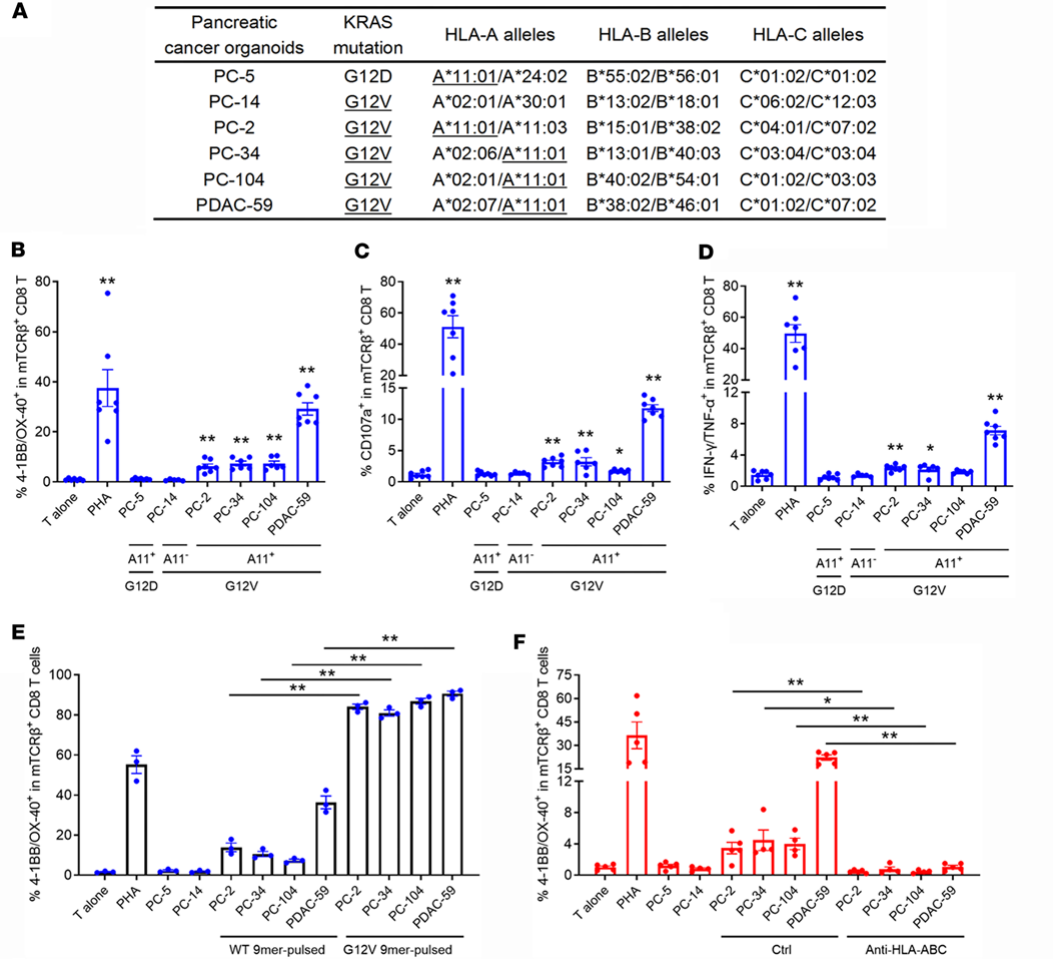
TCR-001–transduced T cells specifically recognized human pancreatic cancer organoids. (**A**) KRAS mutation and HLA profiles of human pancreatic cancer organoids. (**B**) TCR-001–transduced allogeneic T cells were cocultured overnight with human pancreatic cancer organoid cells naturally expressing KRAS G12 mutations or HLA-A*11:01. 4-1BB/OX-40 upregulation was assayed by flow cytometry. (**C** and **D**) TCR-001–transduced allogeneic T cells were cocultured with human pancreatic cancer organoid cells naturally expressing KRAS G12 mutations or HLA-A*11:01 for 5 hours. CD107a upregulation (**C**) and IFN-γ/TNF-α secretion (**D**) were assayed by intracellular cytokine staining. (**E**) TCR-001–transduced allogeneic T cells were cocultured overnight with human pancreatic cancer organoid cells naturally expressing KRAS G12 mutations or HLA-A*11:01 pulsed with 1 μg/mL KRAS WT or G12V 9-mer peptides. 4-1BB/OX-40 upregulation was assayed by flow cytometry. (**F**) TCR-001–transduced allogeneic T cells were cocultured overnight with human pancreatic cancer organoid cells naturally expressing KRAS G12 mutations or HLA-A*11:01. 4-1BB/OX-40 upregulation was assayed by flow cytometry. Anti–human HLA-ABC antibody (20 μg/mL) was used in some groups. Error bars represent SEM of at least 3 biological replicates. Dots indicate biological replicates. *P < 0.05; **P < 0.01 vs. “T alone” group or indicated group. Statistical differences were determined by mixed-effects analysis with Tukey’s multiple-comparison test (**B**–**D** and **F**) or 1-way ANOVA followed by Tukey’s multiple-comparison test (**E**).

**Figure 6:**
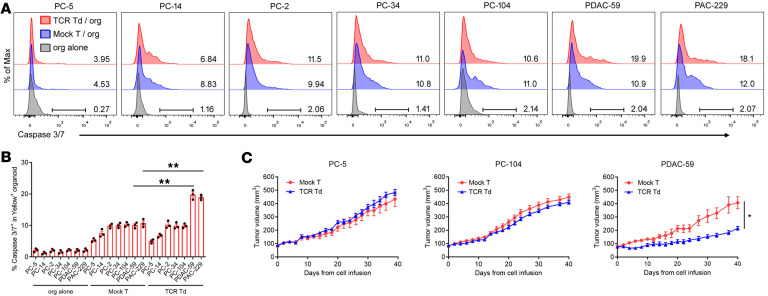
TCR-001–transduced T cells specifically killed only 2 of 5 tested human pancreatic cancer organoids. (**A** and **B**) TCR-001–transduced allogeneic T cells or mock T cells were cocultured with human pancreatic cancer organoid cells naturally expressing the KRAS G12V mutation and HLA-A*11:01 on rat tail collagen–coated plates at an effector/target ratio of 10:1. Human pancreatic cancer organoid cells were labeled with CellTrace Yellow before the coculture. After 24 hours of coculture, tumor cell apoptosis in Yellow^+^ organoid cells was assayed by green fluorescent caspase 3/7 probe using flow cytometry. Representative overlay plots (**A**) and summarized data (**B**) are shown. Numbers in overlay plots indicate the percentage of caspase 3/7^+^ cells among gated Yellow^+^ organoid cells. Error bars represent SEM of 3 biological replicates. Dots indicate biological replicates. **P < 0.01 by 1-way ANOVA test followed by Tukey’s multiple-comparison test. (**C**) Organoid-derived xenografts were established by human pancreatic cancer organoids PC-5, PC-104, and PDAC-59 in NPI immunodeficient mice. After tumor size reached approximately 80 mm^3^, mice were injected with 1 × 10^7^ TCR-001–transduced T cells (TCR Td) or mock T cells and tumor growth was measured (*n* = 6 per group). Error bars represent SEM. *P < 0.05 by 2-tailed, unpaired *t* test.

To evaluate whether TCR-001 can potentially be used to treat pancreatic cancer, we investigated the interaction between TCR-001–transduced allogeneic T cells and human pancreatic cancer organoids. Increasing evidence demonstrates that human pancreatic cancer organoids can recapitulate the features of patient cancer cells and be used for drug screening or studies of the tumor microenvironment (24–26, 29, 36–38). To evaluate specific recognition of TCR-001–transduced T cells on human pancreatic cancer organoids, TCR-001–transduced allogeneic T cells were cocultured with human pancreatic cancer organoids naturally expressing KRAS G12 mutations with or without natural HLA-A*11:01 expression (Figure 5A). The results of 4-1BB/OX-40 upregulation, CD107a upregulation, and IFN-γ/TNF-α secretion showed that TCR-001–transduced allogeneic T cells selectively recognized several human pancreatic cancer organoids with natural KRAS G12V mutation and HLA-A*11:01 expression to different degrees (Figure 5, B–D), which could be due to different levels of antigen presentation in these human pancreatic cancer organoids. In general, though, the recognition of the organoids was weak to modest. However, among these 4 human pancreatic cancer organoids with natural KRAS G12V mutation and HLA-A*11:01 expression, PDAC-59 activated TCR-001–transduced allogeneic T cells to the highest level (Figure 5, B–D). All 4 pancreatic cancer organoids pulsed with KRAS G12V 9-mer peptide greatly activated TCR-001–transduced allogeneic T cells (Figure 5E), indicating sufficient levels of HLA-A*11:01 on these organoids. Furthermore, specific recognition of TCR-001–transduced allogeneic T cells on human pancreatic cancer organoids was dependent on HLA-I (Figure 5F). To evaluate whether TCR-001–transduced allogeneic T cells could specifically kill human pancreatic cancer organoids, TCR-001–transduced allogeneic T cells or mock T cells were cocultured with human pancreatic cancer organoids naturally expressing KRAS G12 mutations with or without natural HLA-A*11:01 expression on rat tail collagen–coated plates. The results showed that TCR-001–transduced allogeneic T cells, but not mock T cells, specifically killed human pancreatic cancer organoid PDAC-59 and PAC-229, but not PC-2, PC-34, and PC-104, all of which express the natural KRAS G12V mutation and HLA-A*11:01, suggesting that while these 3 organoids can be recognized by the TCR-engineered T cells (Figure 5, B–D), the recognition is not sufficient to induce cytolysis (Figure 6, A and B, and Supplemental Figure 8). Notably, similarly to PDAC-59, PAC-229 was strongly recognized by TCR-001–transduced allogeneic T cells (Supplemental Figure 9). To determine whether our observations using the in vitro organoid system could be extended to the in vivo setting, we performed adoptive transfer experiments in immunodeficient mice harboring established patient-derived organoids. The transfer of TCR-001–transduced T cells into the mice could inhibit the growth of PDAC-59 organoid–derived xenografts, but could not control the growth of the negative control PC-5 organoid–derived xenografts, and only had weak effects against the HLA-A*11:01^+^ and KRAS G12V^+^ PC-104 organoid–derived xenografts (Figure 6C), consistent with the in vitro organoid reactivity data.

### IFN-γ priming enhanced the recognition and killing of human pancreatic cancer organoids by TCR-001–transduced T cells.

**Figure 7:**
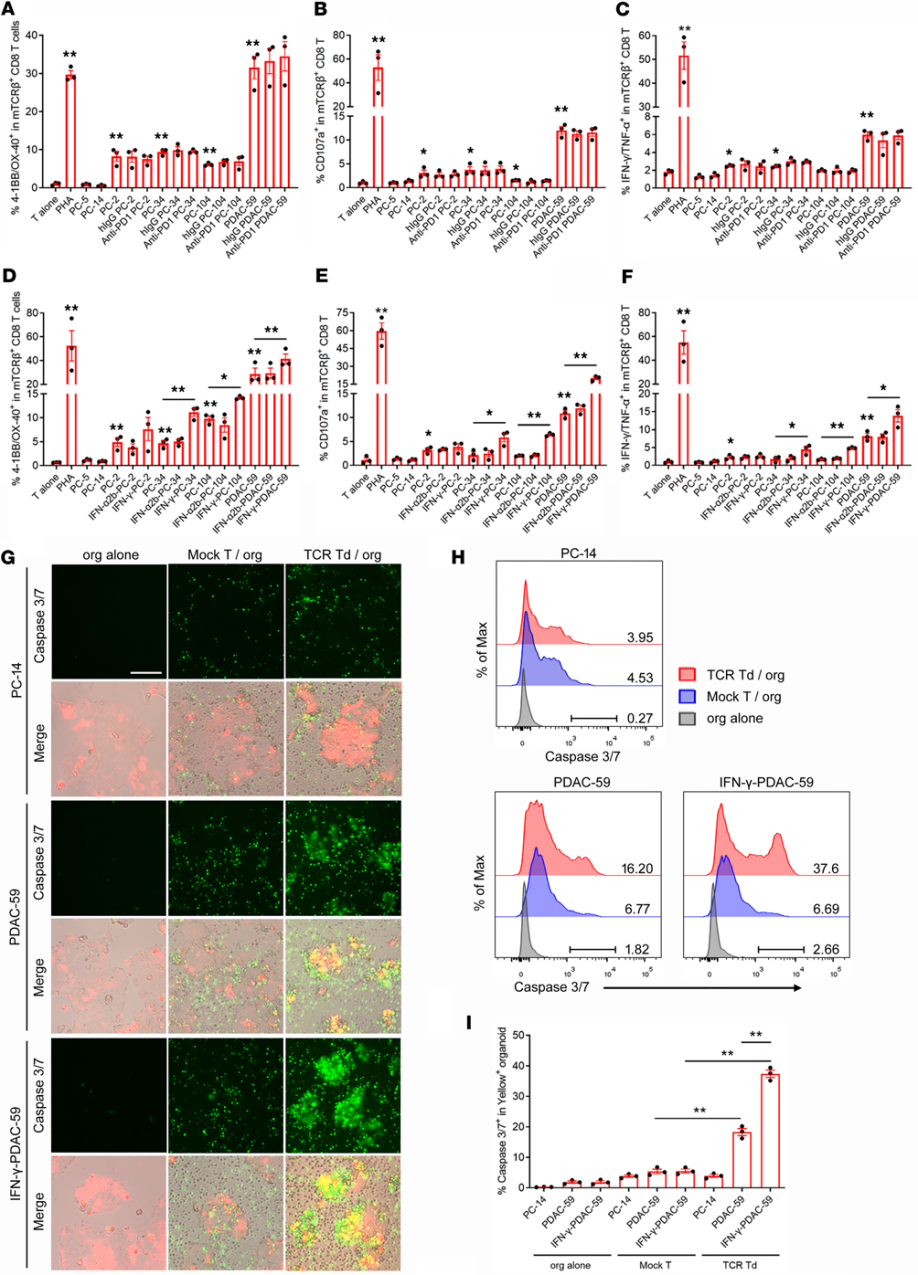
IFN-γ priming enhanced the recognition and killing of human pancreatic cancer organoids by TCR-001–transduced T cells. (**A**–**C**) TCR-001–transduced allogeneic T cells were cocultured with human pancreatic cancer organoid cells naturally expressing KRAS G12 mutations or HLA-A*11:01. In some groups, 10 μg/mL anti–human PD-1 antibody (Beigene) or human IgG (Invitrogen) was added. 4-1BB/OX-40 upregulation (**A**), CD107a upregulation (**B**), and IFN-γ/TNF-α secretion (**C**) were assayed by flow cytometry and intracellular cytokine staining. (**D**–**F**) TCR-001–transduced allogeneic T cells were cocultured with human pancreatic cancer organoid cells naturally expressing KRAS G12 mutations and HLA-A*11:01. In some groups, organoid cells were primed with 100 ng/mL IFN-α2b or 125 ng/ml IFN-γ for 24 hours before the coculture. 4-1BB/OX-40 upregulation (**D**), CD107a upregulation (**E**), and IFN-γ/TNF-α secretion (**F**) were assayed by flow cytometry and intracellular cytokine staining. (**G**) TCR-001–transduced allogeneic T cells or mock T cells were cocultured with human pancreatic cancer organoid cells naturally expressing the KRAS G12V mutation and HLA-A*11:01 at an effector/target ratio of 10:1. Human pancreatic cancer organoid cells were labeled with CellTrace Yellow before the coculture. In some groups, PDAC-59 organoid cells were primed with 125 ng/mL IFN-γ before the coculture. After 24 hours of coculture, tumor cell apoptosis was detected by green fluorescent caspase 3/7 probe. Representative images with caspase 3/7 green fluorescence and merged figures between caspase 3/7 green fluorescence and corresponding bright-field and CellTrace Yellow are shown. Scale bar: 100 μm. (**H** and **I**) Apoptosis in Yellow^+^ organoid cells was assayed by green fluorescent caspase 3/7 probe using flow cytometry. Representative overlay plots (**H**) and summarized data (**I**) are shown. Numbers in overlay plots indicate the percentage of caspase 3/7^+^ cells among gated Yellow^+^ organoid cells. Error bars represent SEM of at least 3 biological replicates. Dots indicate biological replicates. *P < 0.05; **P < 0.01 vs. “T alone” group or indicated group. Statistical differences were determined with 1-way ANOVA followed by Tukey’s multiple-comparison test (**A**–**F** and **I**).

It is well known that pancreatic cancer is one of the most treatment-refractory cancers (39–41). We next investigated the potential of combination therapies with TCR-001–transduced T cells. As shown in Figure 7, A–C, anti–human programmed death 1 (anti–PD-1) antibody did not enhance the reactivity of TCR-001–transduced allogeneic T cells to human pancreatic cancer organoids with natural KRAS G12V mutation and HLA-A*11:01 expression, which might be due to low-level PD-1 expression of TCR-001–transduced allogeneic T cells (data not shown). Appropriate antigen presentation and IFN responses are critical for the recognition of cancer cells by TCR-transduced T cells (42, 43). Thus, human pancreatic cancer organoids next were primed with IFN-α2b or IFN-γ before the coculture with TCR-001–transduced allogeneic T cells, which led to low to modest increases in the expression of HLA-I and PD-L1 (Supplemental Figure 10). The results of 4-1BB/OX-40 upregulation, CD107a upregulation, and IFN-γ/TNF-α secretion showed that IFN-γ priming, but not IFN-α2b priming, enhanced the selective recognition of TCR-001–transduced allogeneic T cells against most of the human pancreatic cancer organoids with natural KRAS G12V mutation and HLA-A*11:01 expression (Figure 7, D–F). Furthermore, TCR-001–transduced allogeneic T cells induced more cell apoptosis of PDAC-59 when primed with IFN-γ compared with when PDAC-59 was not primed with IFN-γ, while TCR-001–transduced allogeneic T cells did not induce cell apoptosis in the negative control organoid PC-14 (Figure 7, G–I), demonstrating that IFN-γ priming enhanced specific killing of human pancreatic cancer organoids by TCR-001–transduced T cells.

## Discussion

Adoptive cell transfer using neoantigen-specific TILs can lead to durable tumor regression in some patients with common epithelial cancers (1, 11, 44–46), but does not work frequently. This is likely due to highly differentiated or exhausted phenotypes of TILs, low frequency of neoantigen-reactive T cells among TILs, passenger but not clonal neoantigen-specific TILs, and cancer cell–intrinsic defects in antigen processing or presentation (9, 11, 31, 47, 48). Adoptive cell transfer using less-differentiated peripheral blood lymphocytes genetically engineered to express allogeneic TCRs targeting oncogenic KRAS may overcome some of these challenges. Indeed, a recent case report showed objective regression of metastatic pancreatic cancer mediated by TCR-gene therapy targeting KRAS G12D (2). Identifying more potentially therapeutic TCRs targeting KRAS neoantigen may benefit numerous patients with KRAS mutation. Furthermore, using patient cancer organoids naturally expressing neoantigen and specific HLA may allow for a deeper preclinical characterization of the therapeutic potential of TCRs.

In this study, we developed a sequential in vitro stimulation approach and identified a total of 2 reactivities against the KRAS G12V mutation from 6 PDAC patients with KRAS G12V mutation and HLA-A*11:01 expression. Among these 2 reactivities against the KRAS G12V mutation, an HLA-A*11:01–restricted TCR was identified and validated to possess high specificity, while an HLA-DRB1*15:01–restricted TCR targeting mutant KRAS G12V was identified from CD4^+^ T cells. Additional characterization of the HLA-DRB1*15:01–restricted KRAS G12V–reactive TCR (e.g., functional avidity, minimal core epitope) remains to be determined. Of note, NetMHCII 2.3 and NetMHCIIpan 4.3e predicted the peptide sequence LVVVGAVGV as the potential core epitope for the KRAS G12V epitope restricted by HLA-DRB1*15:01 (data not shown), a finding that requires confirmation with in vitro studies. Importantly, our identified HLA-A*11:01–restricted mutant KRAS G12V_8–16_–reactive TCR–transduced T cells could specifically recognize all tested human pancreatic cancer organoids that expressed HLA-A*11:01 and KRAS G12V, but only killed some of them. The recognition of human pancreatic cancer organoids by this TCR could be enhanced by IFN-γ priming of organoids. Our study shows that this TCR has therapeutic potential for use in TCR-engineered T cell therapy, but that other therapies that increase IFN-γ signaling in the tumor microenvironment may be required to enhance tumor recognition by the TCR-engineered T cells and increase therapeutic activity.

In our established sequential in vitro stimulation approach, the exposure to GM-CSF plus R848 and LPS was used to enhance antigen-presenting cells among PBMCs to present long peptides to CD4^+^ T cells or cross-present long peptides to CD8^+^ T cells likely through producing high levels of IL-12 p70, and then IL-7 was used to drive the expansion of antigen-specific T cells. Previously, a similar approach could efficiently expand natural tumor-associated antigen Her2- or MUC1-reactive CD8^+^ and CD4^+^ T cells from unvaccinated healthy donor or patient PBMCs (34). Both KRAS G12V neoantigen–reactive CD8^+^ T cells and CD4^+^ T cells could be identified by using our approach. Importantly, our identified KRAS G12V neoantigen–reactive CD8^+^ T cells or CD4^+^ T cells were not reactive to WT KRAS peptide–pulsed autologous DCs. Our identified KRAS G12V neoantigen–reactive CD8^+^ T cells and CD4^+^ T cells were derived from PBMCs from approximately 1 year after surgery and PBMCs from before surgery or about 9 months after surgery, respectively, indicating that PBMCs from both before and after surgery could be used to screen for KRAS neoantigen–reactive T cells. In ongoing research, we are trying to identify additional TCRs targeting KRAS mutations in human pancreatic cancer patients with other KRAS mutations or HLA expression using our sequential in vitro stimulation approach.

The identified HLA-A*11:01–restricted mutant KRAS G12V_8–16_–reactive TCR had a different sequence from previously reported HLA-A*11:01–restricted KRAS G12V–reactive TCRs (8, 12, 16). Our TCR bound to the 9-mer HLA-A*11:01–restricted mutant KRAS G12V_8–16_ epitope, but not the 10-mer KRAS G12V_7–16_ peptide that is also predicted to bind to HLA-A*11:01 (data not shown). Furthermore, our TCR recognized mutant KRAS G12V_8–16_, but not KRAS WT_8–16_ peptide, which suggests that our TCR should not recognize normal cells and potentially can be used as an “off-the-shelf” reagent for cancer immunotherapy. Also, CD4^+^ T cells that expressed our TCR did not recognize tumor cells, indicating that our TCR is CD8-coreceptor dependent and not a super-high-affinity TCR. Considering the second rank of the KRAS G12V mutation in all KRAS-mutated cancers (24%) and high prevalence of HLA-A*11:01 in Chinese and Asian populations of the United States (both >30%) (13), our HLA-A*11:01–restricted mutant KRAS G12V_8–16_–reactive TCR could be used in TCR-based therapies for a large number of patients with KRAS-mutated cancer within these 2 populations (>7.2%).

In addition to specific recognition of cognate antigen in tumor cells by TCR-transduced T cells, it is critical to validate that potentially therapeutic TCR-transduced T cells can kill patient tumor cells. However, most investigators have used tumor cell lines and not patient primary tumor cells to validate the killing of tumor target cells by mutant KRAS–specific TCR–transduced T cells in vitro or in vivo (6, 7, 12, 14, 15). In this study, we used patient pancreatic cancer organoids to validate the recognition and killing by our identified HLA-A*11:01–restricted KRAS G12V–reactive TCR–transduced T cells. Using patient pancreatic cancer organoids with natural KRAS G12V mutation and HLA-A*11:01 expression, our identified HLA-A*11:01–restricted KRAS G12V–reactive TCR demonstrated the ability to specifically recognize all tested patient cancer organoids to different degrees, but only kill some of them, which indicates that individual patients likely will respond differently to therapy with TCR-transduced T cells, potentially due to different levels of antigen presentation in tumor cells. Our in vivo data, consistent with the in vitro organoid reactivity data, demonstrated that the TCR-transduced T cells could inhibit the growth of specific organoid-derived xenografts, but not eradicate the tumor, which might have potential clinical implications. Further analysis with whole-exome sequencing and RNA-sequencing data of these patient cancer organoids may help reveal the underlying mechanisms behind the varied reactivity. Notably, a previous study using immunopeptidomics and targeted mass spectrometry demonstrated that there was a lower abundance of KRAS G12V_8–16_ 9-mer peptide per cell compared with the KRAS G12V_7–16_ 10-mer peptide in the context of HLA-A*11:01 on the surface of HLA-A*11:01–engineered natural KRAS G12V^+^ CORL23 tumor cells (6). However, other studies detected only the KRAS G12V_7–16_ 10-mer peptide and not KRAS G12V_8–16_ 9-mer peptides from primary tumor tissues (49), natural HLA-A*11:01^+^ KRAS G12V^+^ Colo668 tumor cells (49), or HLA-A*11:01–engineered natural KRAS G12V^+^ SW620 tumor cells (7). The discrepancy between these studies could be due to the different tumor samples and/or the differences in the sensitivity of the technologies used to detect the KRAS peptides. Nonetheless, these studies indicate that the amount of KRAS G12V_8–16_ 9-mer peptides presented by tumors may be relatively low, which could at least in part contribute to the weak to modest recognition of the TCR against some of the organoids observed in our study. It will be interesting to determine whether the degree of in vitro tumor organoid recognition and killing by TCR-transduced T cells is correlated with clinical response to TCR therapy since this may impact how future patients are screened for eligibility. We also found that IFN-γ priming of patient pancreatic cancer organoids could efficiently enhance the specific recognition and killing of patient pancreatic cancer organoids by our identified HLA-A*11:01–restricted KRAS G12V–reactive TCR-transduced T cells, which highlights that patient cancer organoids can be used to test potential combination treatment approaches for TCR-gene therapy. IFN-γ priming of patient cancer organoids enhanced HLA-I expression that likely led to the enhanced reactivity of the KRAS G12V–reactive TCR (50, 51), but the exact mechanisms require further investigation. Importantly, we currently are performing a clinical trial (ClinicalTrials.gov NCT04146298) to evaluate the safety and efficacy of our identified HLA-A*11:01–restricted KRAS G12V_8–16_–specific TCR in a TCR-gene therapy trial for patients with advanced pancreatic cancer.

## Methods

### Sex as a biological variable.

This study involved male and female patients with pancreatic cancer. For murine studies, 6- to 8-week-old male NPI (NOD.*Prkdc^–/–^* *Il2rg^–/–^*) immunodeficient mice were used. Sex was not considered as a biological variable, as the incidence and outcome of human pancreatic cancer is similar for both sexes.

### Patients information.

Peripheral blood samples were obtained from 7 patients with PDAC. Three patients were diagnosed as stage I and 4 as stage II. Patients were treatment naive, except Pt.004 and Pt.005 who had received neoadjuvant chemotherapy. The clinical characteristics of the individuals are summarized in Supplemental Table 4. KRAS mutation and HLA-ABC profiles of the individuals were detected by whole-exome sequencing and are shown in Supplemental Table 5.

### Whole-exome sequencing and data processing.

Whole-exome sequencing was performed on DNA extracted from fresh tumor tissue and matched peripheral blood, as described previously (52). The DNA libraries were sequenced on the Illumina HiSeq platform aiming for 200× coverage for blood as well as tissue, and 150-bp paired-end reads were generated. DNA sequencing data were processed according to the Genome Analysis Toolkit best practices workflow and somatic mutations were called as described previously (52).

### Pancreatic cancer cell lines and organoid cell culture.

Human pancreatic cancer cell lines, including BxPC-3, Panc-1, Mia-Paca2, and CFPAC-1 (all from ATCC) were cultured in DMEM (Corning) containing 10% fetal bovine serum (FBS) (Gemini), 100 U/mL penicillin (GIBCO), and 100 μg/mL streptomycin (GIBCO). Human pancreatic cancer cell lines in addition to Panc-1 were transfected with lentiviral particles produced by lentiviral vector plasmid CDH-EF1α-HLA-A*11:01-P2A-β2m-PGK-PuroR-WPRE (Wuhan MiaolingBio, plasmid map shown in Supplemental Figure 6) and packaging plasmids including LP1, LP2, and VSV-G (Wuhan MiaolingBio) and stably screened using 2 μg/mL puromycin (Amresco) to overexpress HLA-A*11:01.

The following culture media were used for pancreatic cancer organoid cells culture: basic medium (advanced DMEM/F12 [GIBCO], 10 mM HEPES [Sangon], 1× GlutaMax [GIBCO], 100 μg/mL Primocin [Invivogen], 1× penicillin/streptomycin solution [GIBCO]) and complete medium (advanced DMEM/F12, 10 mM HEPES, 1× GlutaMax, 100 μg/mL Primocin, 1× penicillin/streptomycin solution, 500 nM A83-01 [Tocris], 10 μM Y-27632 [Selleck], 1.56 mM *N*-acetylcysteine [Sigma-Aldrich], 10 mM nicotinamide [Sigma-Aldrich], 10 ng/ml FGF10 [Peprotech], 1× B27 supplement [GIBCO], 10 μM forskolin [Selleck], 30% Wnt3A-conditioned medium [in-house], 2% R-spondin–conditioned medium [in-house], 4% Noggin-conditioned medium [in-house]). Pancreatic cancer organoid cells used here had been established previously by our laboratory (38) and are maintained on rat tail collagen–coated flat culture plates or suspension plates with Matrigel (Corning, 356231) for long-term culture. The media used for organoid cells cryopreservation were composed of the corresponding complete culture medium (90%) and 10% DMSO. The established organoid cells were routinely tested for mycoplasma contamination.

### Human PBMC isolation.

Human PBMCs were isolated from whole blood of patients or healthy donors by density gradient centrifugation using Lymphoprep (Alere Technologies), as described previously with minor modifications (53) and cryopreserved for further analysis or use.

### Human DC culture.

Human DCs were cultured as described previously, with minor modification (21). PBMCs were thawed in warmed RPMI-1640 medium (Corning) containing 10% FBS and 0.01 mg/mL DNase I and centrifuged at 300*g* for 5 minutes. Cell pellets were resuspended with AIM-V medium (Invitrogen) and allowed to rest on tissue culture–treated 6-well plates (approximately 1 × 10^6^ cells/cm^2^) for 90 minutes. Nonadherent cells were removed and the adherent cells were vigorously washed twice with RPMI-1640 and continued to rest for 60 minutes with AIM-V medium. After that, nonadherent cells were removed again and adherent cells were allowed to culture for 3 days in DC media (RPMI-1640 plus 5% heat-inactivated commercial human AB serum [Gemini], 100 U/mL penicillin [GIBCO], 100 μg/mL streptomycin [GIBCO], 0.25 μg/mL amphotericin B [Sangon], 1× GlutaMax [GIBCO], 25 mM HEPES [Sangon], 800 IU/mL GM-CSF [Novoprotein], and 40 ng/mL IL-4 [Novoprotein]). Media were replenished with fresh DC media (800 IU/mL GM-CSF and 40 ng/mL IL-4) on day 3. Cells were harvested for screening or frozen for future use on day 5.

### Patient PBMC-T culture.

Patient PBMC-Ts were cultured as described previously (34), with minor modifications (as depicted in Figure 1A). On day 0, thawed PBMCs were resuspended in T cell media (AIM-V medium plus 2% heat-inactivated commercial human AB serum, 100 U/mL penicillin, 100 μg/mL streptomycin, 0.25 μg/ml amphotericin B, and 1× GlutaMax) supplemented with 50 ng/mL rhGM-CSF (Novoprotein), and 3 × 10^6^ PBMCs were plated at 0.5 mL per well in 48-well plates (Costar Corning). On day 1 of culture, PBMCs were pulsed with 50 μg/mL HPLC-purified KRAS G12V 24-mer peptide (amino acid sequence: MTEYKLVVVGAVGVGKSALTIQLI), and exposed to 3 μg/mL R848 (Sigma-Aldrich) 4 hours later, and then to 5 ng/mL LPS (Sigma-Aldrich) 1 hour later. On day 2 of culture, cells were harvested, with cell detachment facilitated by incubating with PBS containing 5 mM EDTA for 5 minutes. Cells were centrifuged, washed again in PBS, resuspended in T cell media containing 50 ng/mL rhIL-7 (Novoprotein) to 12 times the initial volume, and plated at 1 mL per well in fresh 48-well plates. Then, every 3–5 days, half of the culture media were replaced with T cell media containing 50 ng/mL rhIL-7 and cells were split into fresh 48-well plates as necessary. After 10–14 days from the start of culture, PBMC-Ts were harvested for detecting the presence of KRAS G12V–reactive T cells or frozen for future use. In some experiments, PBMC-Ts were expanded with autologous PBMCs pulsed with 50 μg/mL KRAS G12V 24-mer peptide at a ratio of 1:1 for approximately 10–14 days.

### Cytometric bead array assay of IL-12 p70.

The concentrations of cytokine IL-12 p70 in culture supernatants were measured by cytometric bead array according to the manufacturer’s protocol (BD Biosciences). The human IL-12 p70 Flex Set (Bead E5) (BD Biosciences) was used for detection of single-cytokine IL-12 p70. The samples were run and FACS data were collected using a BD LSR II cytometer (BD Biosciences) and analyzed using FlowJo software (TreeStar).

### Coculture assays: IFN-γ ELISPOT assays and flow cytometry to assess T cell activation of PBMC-Ts.

For assay of PBMC-T activation, approximately 1 × 10^5^ T cells were seeded per well of a precoated 96-well IFN-γ ELISPOT plate (Dakewe). The cells were stimulated with 10 μg/mL of the HPLC-purified KRAS WT or G12V 24-mer peptide–pulsed allogeneic or autologous DC (3 × 10^4^ to 5 × 10^4^ cells per well) in 50:50 media (complete media and AIM-V media at a ratio of 1:1) without additional cytokine. After overnight coculture (18–24 hours), the cells were harvested and transferred from the ELISPOT plate into a round-bottom 96-well plate for flow cytometry staining and analysis. Complete media consisted of RPMI-1640 supplemented with 10% heat-inactivated commercial human AB serum, 100 U/mL penicillin, 100 μg/mL streptomycin, 0.25 μg/mL amphotericin B, 25 mM HEPES, and 1× GlutaMax.

Then IFN-γ ELISPOT assay was performed according to the manufacture’s protocol using an IFN-γ ELISPOT kit (Dakewe). Briefly, the plates were incubated with deionized water for 10 minutes at 4°C and washed before the addition of the diluted detection antibody (1:100 dilution) and then incubated for 1 hour at 37°C. After washing of the plates, streptavidin-HRP (1:100 dilution) was added and incubated at 37°C for another 1 hour. After washing of the plates, 3-amino-9-ethylcarbazole (AEC) solution mix was then added to each well, and the plates were left in the dark for approximately 10–20 minutes at room temperature. The reaction was stopped by rinsing thoroughly with cold deionized water. The ELISPOT plates were scanned and counted using an ImmunoSpot plate reader and associated software (Cellular Technology Limited).

T cell activation was mainly assessed by flow cytometry for upregulation of the markers OX-40 (CD134) and 4-1BB (CD137) for both activated CD4^+^ and CD8^+^ T cells. See Supplemental Table 6 for details on all antibodies and other reagents. Briefly, cells harvested from the ELISPOT plate were pelleted in a round-bottom 96-well plate and resuspended with FACS buffer (PBS containing 1% FBS and 2 mM EDTA) for flow cytometry as follows.

### Flow cytometry.

For preparation of cells for flow cytometry, cells were harvested, and then resuspended in FACS buffer and incubated with titrated fluorochrome-conjugated antibodies for approximately 30 minutes at 4°C and in the dark. For KRAS G12V 9-mer–HLA-A*11:01 tetramer staining, cells were stained with fluorochrome-conjugated (PE or APC) tetramer (prepared by Shanghai Polaris Biology Co., Ltd.) for 10 minutes and then incubated with titrated fluorochrome-conjugated antibodies for approximately 20 minutes. Cells were then washed with FACS buffer and resuspended with FACS buffer followed by data acquisition with either the BD LSR II or FACSAria Fusion flow cytometer (BD Biosciences). Flow cytometry data were analyzed using FlowJo software.

### T cell sorting and expansion.

Tetramer^+^ CD8^+^ T cells were purified using a Sony SH800S cell sorter based on tetramer expression in CD8^+^ T cells for rapid in vitro expansion using excess irradiated (50 Gy) allogeneic feeder PBMCs pooled from 2 different donors in 50:50 media supplemented with 30 ng/mL anti-CD3 antibody (OKT3, BioLegend) and 3,000 IU/mL IL-2 (Novoprotein), as described previously (21). After approximately 2 weeks, T cells were used in coculture assays or cryopreserved for further analysis.

### HLA restriction assays of KRAS G12V–specific CD4^+^ T cells from PBMC-Ts.

HLA-DRB1*15:01+HLA-DRA*01:01 or HLA-DRB1*11:01+HLA-DRA*01:01 was synthesized and cloned into lentiviral vector plasmids (Wuhan MiaolingBio, plasmid map shown in Supplemental Figure 4). Cos7 cells (ATCC) were plated in a flat-bottom 96-well plate (2.5 × 10^4^ cells/well) and transfected the following day with plasmids encoding HLA-DRB1*15:01+HLA-DRA*01:01 or HLA-DRB1*11:01+HLA-DRA*01:01 (150 ng/well each) using Lipofectamine 3000 (Invitrogen) transfection reagent (0.5 μL/well). The next day, transiently transfected cells were pulsed with 10 μg/mL KRAS WT or G12V 24-mer peptide for 2 hours, washed twice in 50:50 media, and cocultured overnight (18–24 hours) with sorted CD4^+^ T cells from Pt.004 PBMC-Ts (1 × 10^5^/well). 4-1BB/OX-40 expression in CD4^+^ T cells was assayed by flow cytometry, as described above.

### Bulk transcriptomic TCR sequencing of tetramer^+^ CD8^+^ T cells.

Tetramer^+^ CD8^+^ T cells were purified using a Sony SH800S cell sorter based on tetramer expression in CD8^+^ T cells for bulk TCR sequencing. Sorted tetramer^+^ CD8^+^ T cells (approximately 1 × 10^6^ cells) were washed with PBS once and lysed with 1 mL TRIzol (Invitrogen). Total RNA was extracted by RNeasy Mini Kit (Qiagen) and reverse transcribed into cDNA by SuperScript II (Invitrogen) and TruSeq RNA sample preparation kit. Then, the sample was used to perform transcriptomic TCR sequencing by Agilent DNA-1000 in an Agilent Technologies 2100 Bioanalyzer. Acquired clean data were analyzed for TCR assembly and splicing by MiXCR software (54) and analyzed for TCR frequency and repertoire by VDJ tools/tcR software (55).

### Construction of full-length TCR–expressing vector.

The full-length KRAS G12V–reactive TCR was constructed by fusing TRB V-D-J–encoding sequences and TRA V-J–encoding sequences to the modified murine TRB and TRA constant chains, as previously described (21, 35). Use of the murine TCR constant regions promotes pairing of the introduced TCR and facilitates identification of the transduced T cells by flow cytometry using anti–mouse TCR β-chain antibodies (BioLegend). The sequences for the TCR β and α chains were linked together with a furin SGSG P2A linker, and then codon optimized, synthesized, and cloned into the lentiviral plasmid (as shown in Supplemental Figure 5) by OBIO Technology (Shanghai) Corp., Ltd. Lentiviral particles for this TCR was produced by OBIO Technology (Shanghai) Corp., Ltd by transient transfection of 293T cells (ATCC) with plasmids encoding gag/pol, Rev, envelope VSV-G, and this TCR. Supernatants were harvested and TCR-expressing lentiviral particles were purified and titrated.

### Preparation of TCR-transduced T cells.

Pan T cells were purified from thawed healthy donor PBMCs by pan T cell isolation kit (human, Miltenyi Biotec) and then T cells (2 × 10^6^/mL, 1 mL per well of 24-well plate) were stimulated with T Cell TransAct (Miltenyi Biotec) in 50:50 media containing 5% heat-inactivated human AB serum and 300 IU/mL IL-2 (Novoprotein). After 36–48 hours, the activated T cells (5 × 10^5^/mL, 2 mL per well of 24-well plate) were transfected with the lentiviral particles encoding the TCR for transduction. The following day, the cells were transferred to a G-Rex 24-well plate (Wilson Wolf) and further cultured in the above culture media. At approximately 10–14 days after the initial stimulation, aliquots of the cells were cryopreserved for further analysis.

### Assays of specific recognition of endogenously processed and presented antigen by TCR-transduced T cells.

The KRAS G12V full-length gene or KRAS WT full-length gene was synthesized and cloned into pcDNA3.1 vector plasmids (GenScript, plasmid map shown in Supplemental Figure 7). Cos7 cells were plated in a flat-bottom 96-well plate (2.5 × 10^4^ cells/well) and transfected the following day with both plasmid encoding HLA-A*11:01+β2m (Wuhan MiaolingBio, plasmid map shown in Supplemental Figure 6) and plasmid encoding the KRAS G12V full-length gene or KRAS WT full-length gene (150 ng/well each) using Lipofectamine 3000 transfection reagent (0.5 μL/well). The next day, transiently transfected Cos7 cells were washed twice in 50:50 media, and cocultured overnight (18–24 hours) with TCR-transduced T cells (1 × 10^5^/well). 4-1BB/OX-40 expression in T cells was assayed by flow cytometry, as described as follows.

### Coculture assays: flow cytometry and intracellular cytokine staining to assess T cell activation of TCR-transduced T cells by PDAC cancer cells.

For assay of TCR-transduced T cell activation by PDAC cell lines or organoid cells, approximately 1 × 10^5^ TCR-transduced T cells were seeded per well of a 96-well flat plate. The cells were cocultured with various concentrations of the HPLC-purified KRAS peptide–pulsed pancreatic cancer cell lines or pancreatic cancer organoid cells (5 × 10^4^ cells per well) in 50:50 media without additional cytokine. After overnight coculture (18–24 hours), the cells were harvested and transferred into a round-bottom 96-well plate for flow cytometry staining and analysis. T cell activation was mainly assessed by flow cytometry for upregulation of the markers OX-40 (CD134) and 4-1BB (CD137) for both activated CD4^+^ and CD8^+^ T cells. Briefly, cells harvested were pelleted in a round-bottom 96-well plate and resuspended with FACS buffer for flow cytometry.

For intracellular cytokine staining, approximately 1 × 10^5^ TCR-transduced T cells were seeded per well of a 96-well round bottom plate. The cells were cocultured with various concentrations of the HPLC-purified KRAS peptide–pulsed pancreatic cancer cell lines or pancreatic cancer organoid cells (5 × 10^4^ cells per well). The cells were stimulated with 5 μg/mL phytohemagglutinin (PHA) as positive control. PE-conjugated anti-CD107a antibody (H4A3, BioLegend), brefeldin A (BioLegend), and monensin (BioLegend) were added at the onset of stimulation. After 5 hours of stimulation in an incubator at 37°C and 5% CO_2_, the cells were transferred to a 96-well round-bottom plate. Cells were washed once with 200 μL of FACS buffer, and then stained with PE-Cy7–conjugated anti-CD8 antibody (SK1), Pacific Blue–conjugated anti-CD3 antibody (UCHT1), APC/Fire 750–conjugated anti-CD4 antibody (SK3), and FITC-conjugated anti-mTCRβ antibody (H57-597) (all from BioLegend). The cells were incubated in the dark at 4°C for 30 minutes and then washed once with 200 μL of FACS buffer. Cells were then incubated in fixation buffer (BioLegend) in the dark at room temperature for 20 minutes. Cells were washed once with 200 μL of FACS buffer, and then twice with 200 μL of permeabilization buffer (BioLegend). The cells were stained with APC-conjugated anti–IFN-γ antibody (B27) and PerCp/Cy5.5-conjugated anti–TNF-α antibody (MAb11) (all from BioLegend). After a 30-minute incubation in the dark at room temperature, cells were washed once with 200 μL of permeabilization buffer. The cells were resuspended in FACS buffer and analyzed on a BD FACSAria Fusion flow cytometer.

In some experiments, 10 μg/mL anti–human PD-1 antibody (Beigene) or human IgG (Invitrogen) was added during the coculture, or pancreatic cancer organoid cells were primed with 100 ng/mL IFN-α2b or 125 ng/mL IFN-γ for 24 hours before the coculture.

### Assays of the killing of pancreatic cancer organoid cells by TCR-transduced T cells.

To assay the killing of organoid cells by TCR-transduced T cells, organoid cells were harvested, dissociated into single cells, and stained with 5 μM CellTrace Yellow (Invitrogen, C34573) according to the manufacturer’s protocol. Organoid cells (1 × 10^4^ per well) were cocultured with mock T or TCR-transduced T cells at a ratio of 1:10 in rat tail collagen–coated 96-well flat-bottom plates with coculture media (organoid complete media/50:50 media = 1:1 v/v). In some experiments, organoid cells (1 × 10^4^ per well) were previously cultured in rat tail collagen–coated 96-well flat-bottom plates without or with 125 ng/mL IFN-γ for 24 hours and then IFN-γ–primed organoid cells were washed with organoid complete media twice, culture medium was changed to coculture media (0.1 mL/well), and mock T cells or TCR-transduced T cells (1 × 10^5^ cells/well, 0.1 mL/well) were added. At the start of coculture, caspase 3/7 green probe (Invitrogen, C10723) was added at a 1:2000 dilution to visualize cell apoptosis. After 24 hours of coculture, images were acquired by inverted microscope (Olympus IX73) to observe the apoptosis of organoid cells induced by TCR-transduced T cells and then organoid cells were dissociated into single cells for flow cytometry assay for caspase 3/7 expression in CellTrace Yellow^+^ organoid cells.

### Organoid-derived xenograft treatment by adoptive transfer of TCR-transduced T cells.

Pancreatic cancer organoid cells were injected into NPI (NOD.*Prkdc^–/–^* *Il2rg^–/–^*) immunodeficient mice (56) lacking T cells, B cells, and NK cells (Beijing IDMO Co.Ltd.) subcutaneously (2 × 10^6^ per mouse in 100 μL FBS-free medium and 100 μL Matrigel (Corning, 354234). After tumor size reached approximately 80 mm^3^, 1 × 10^7^ TCR-001–transduced T cells or mock T cells were injected intravenously, following by daily intraperitoneal 200,000 IU IL-2 injection for 3 days. Tumor growth was measured by caliper every 2–4 days, and tumor volume (mm^3^) was calculated by length × width^2^/2.

### Statistics.

Related quantitative data were analyzed using Prism version 8.0.2 software (GraphPad Software, Inc.). Data are shown as mean ± SEM in related graphs. Statistical comparisons between 2 groups were analyzed with a 2-tailed, unpaired *t* test and a *P* value of less than 0.05 was considered statistically significant. For statistical comparisons of more than 2 groups, 1-way ANOVA test followed by a post hoc analysis (Tukey’s multiple-comparison test) or mixed-effects analysis with Tukey’s multiple-comparison test was used and a *P* value of less than 0.05 was considered statistically significant.

### Study approval.

All patients were enrolled in Shanghai Changhai Hospital Ethics Committee–approved research protocol 2019-034. All blood samples were obtained after written informed consent was provided by the patients in the study. All animal studies (including the mice euthanasia procedure) were done in compliance with the regulations and guidelines of Changhai Hospital of Shanghai institutional animal care and were conducted according to the AAALAC and the IACUC guidelines.

### Data availability statement.

All data related to the findings in this study are available in the main text, the supplemental material, or the Supporting Data Values file. Any additional information required to reanalyze the data reported in this paper is available from the corresponding author contact upon request.

## Author contributions

GJ, ET, and DG conceived and supervised the study. XX, SG, and ZC designed the experiments. XX, HG, XS, XY, and HW performed the experiments. CZ, YS, and YL helped the experiments and provided technical support. SG, BL, and LZ provided all the clinical information. XX, WJ, KZ, ZS, and PC analyzed all the data. XX, GJ, ET, DG, and BQ prepared the manuscript. All authors reviewed the results and approved the final version of the manuscript.

## Supplementary Material

Supplemental data

Supporting data values

## Version 1 01/23/2025

Electronic publication
