# Supplementary material for: Identification and validation of a T cell receptor targeting KRAS G12V in HLA-A*11:01 pancreatic cancer patients
Source: JCI Insight. 2025 Jan 23;10(2):e181873. doi: 10.1172/jci.insight.181873 (PMC11790028; doi:10.1172/jci.insight.181873)
Supplement: Supplemental data [file jciinsight-10-181873-s021.pdf]

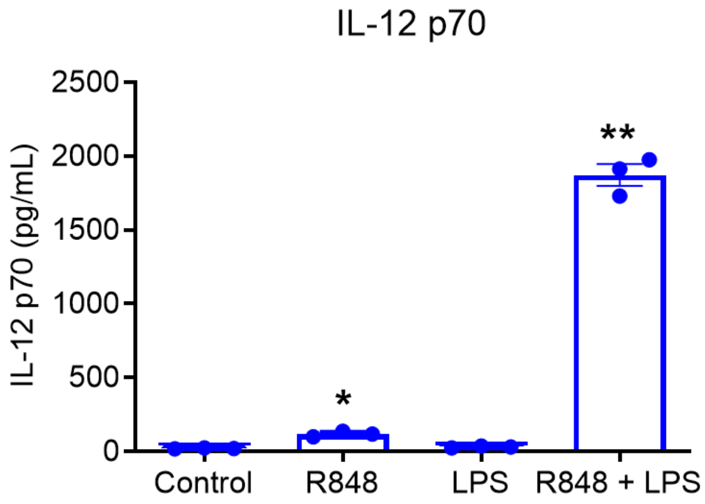

Supplemental Figure 1. IL-12 p70 production of healthy donor PBMC after various TLR agonists stimulation

Thawed healthy donor PBMC were cultured overnight in T cell media containing 50 ng/mL rhGM-CSF. Then cells were stimulated with 3 μg/mL R848, 5 ng/mL LPS or 3 ug/mL R848 + 5 ng/mL LPS (R848 was added and one hour later LPS was added). After 24 hours of LPS stimulation, cell supernatant was harvested and IL-12 p70 was detected by CBA assay. There were three replicate wells in each group. "*", $p < 0.05$, versus "Control" group; "**", $p < 0.01$, versus "Control" group. Statistical differences were determined with one-way ANOVA test followed by a post hoc analysis (Tukey's multiple comparison test).

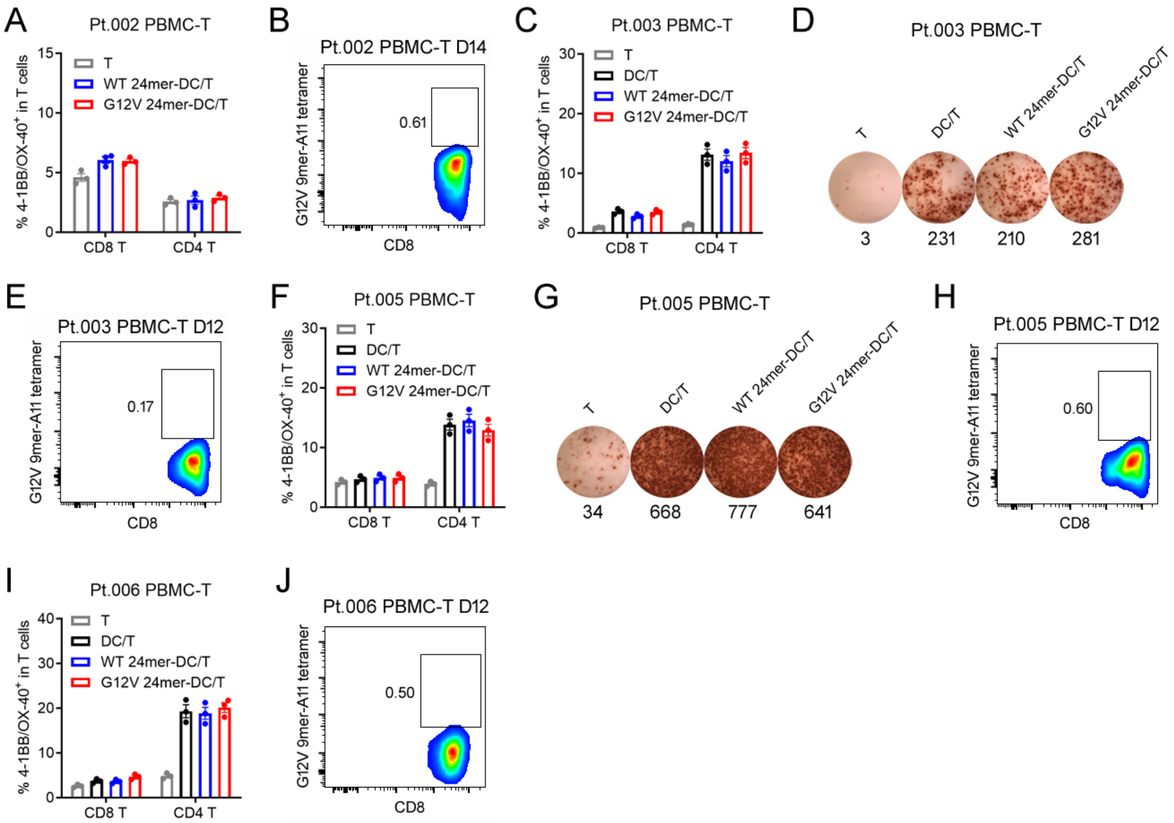

Supplemental Figure 2. No mutant KRAS G12V reactive T cells identified in Pt.002, Pt.003, Pt.005 and Pt.006

(A, C, D, F, G) PBMC-Ts from Pt.002 PBMC before surgery (A), Pt.003 PBMC before surgery (C, D) or Pt.005 PBMC before surgery (F, G) were cultured, and the reactivity was tested against allogeneic HLA-A*11: 01-expressing DC (from Pt.007) pulsed with KRAS WT or G12V 24mer peptides by 4-1BB/OX-40 upregulation (A, C, F) and/or ELISPOT IFN-γ secretion (D, G) assays. (I) PBMC-Ts from Pt.006 PBMC before surgery were cultured, and the reactivity was tested against autologous DC pulsed with KRAS WT or G12V 24mer peptides by 4-1BB/OX-40 upregulation assay. (B, E, H, J) PBMC-Ts from Pt.002 (B), Pt.003 (E), Pt.005 (H), and Pt.006 (J) were stained with KRAS G12V 9mer-A11 tetramer. The numbers in plots indicated the percentage of tetramer$^+$ CD8 T cells among CD8 T cells. Mean ± SEM from three technical replicates are shown.

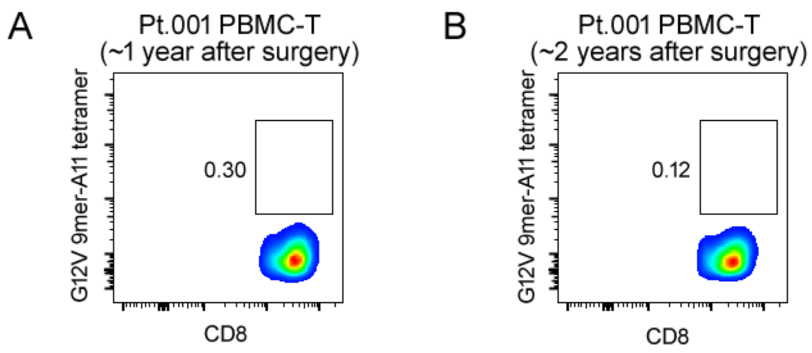

Supplemental Figure 3. No mutant KRAS G12V reactive T cells identified from other two batches of PBMC-Ts in Pt.001

PBMC-Ts (Batch 2) from Pt.001 PBMC from about 1 year after surgery (A) or PBMC-Ts (Batch 3) from Pt.001 PBMC from about 2 years after surgery (B) were stained with KRAS G12V 9mer-A11 tetramer. The numbers in plots indicated the percentage of tetramer$^+$ CD8 T cells among CD8 T cells.

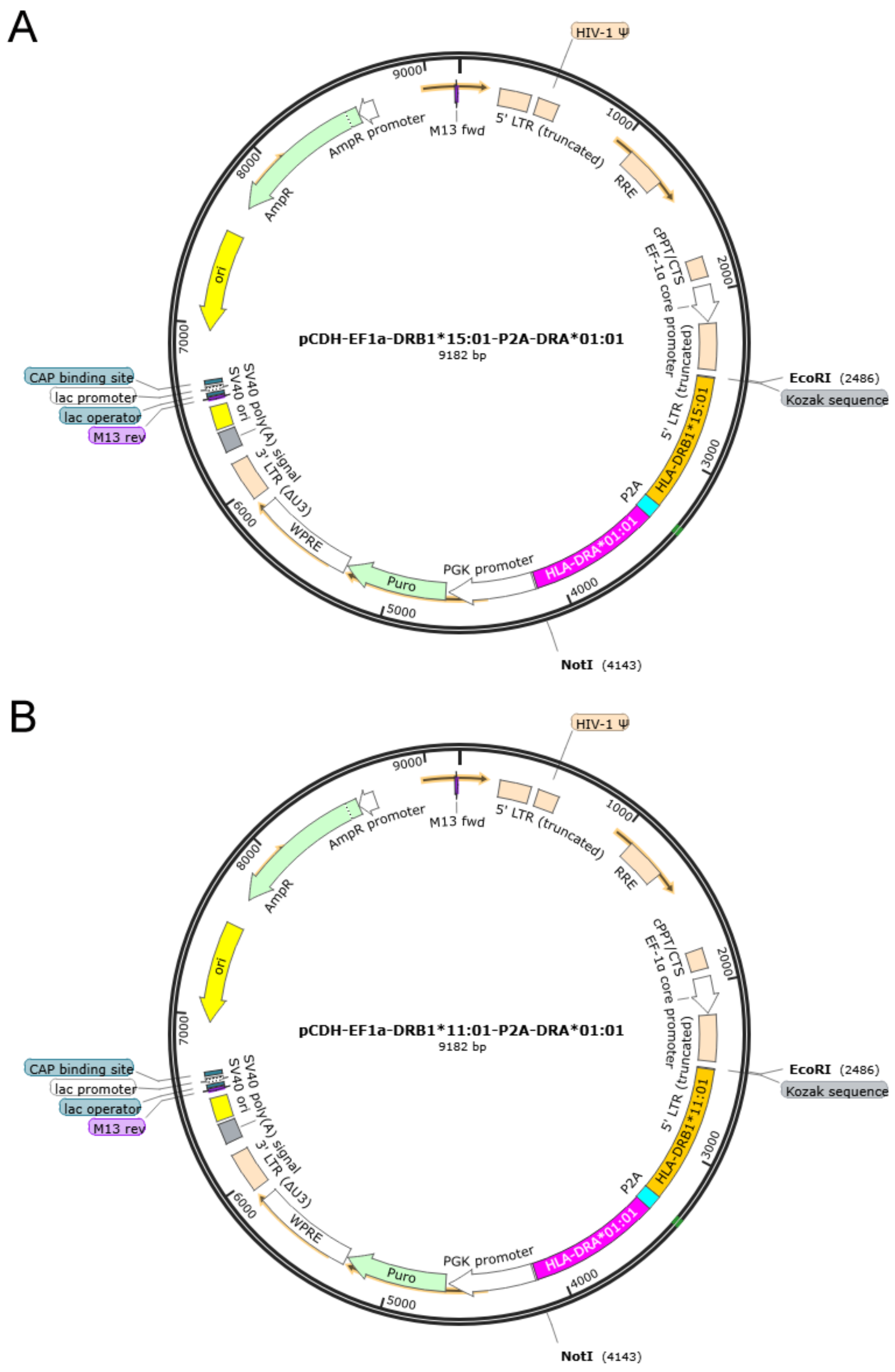

Supplemental Figure 4. The maps of lentiviral vectors expressing human HLA-DRB1*15:01 and HLA-DRA*01:01 (A) or HLA-DRB1*11:01 and HLA-DRA*01:01 (B)

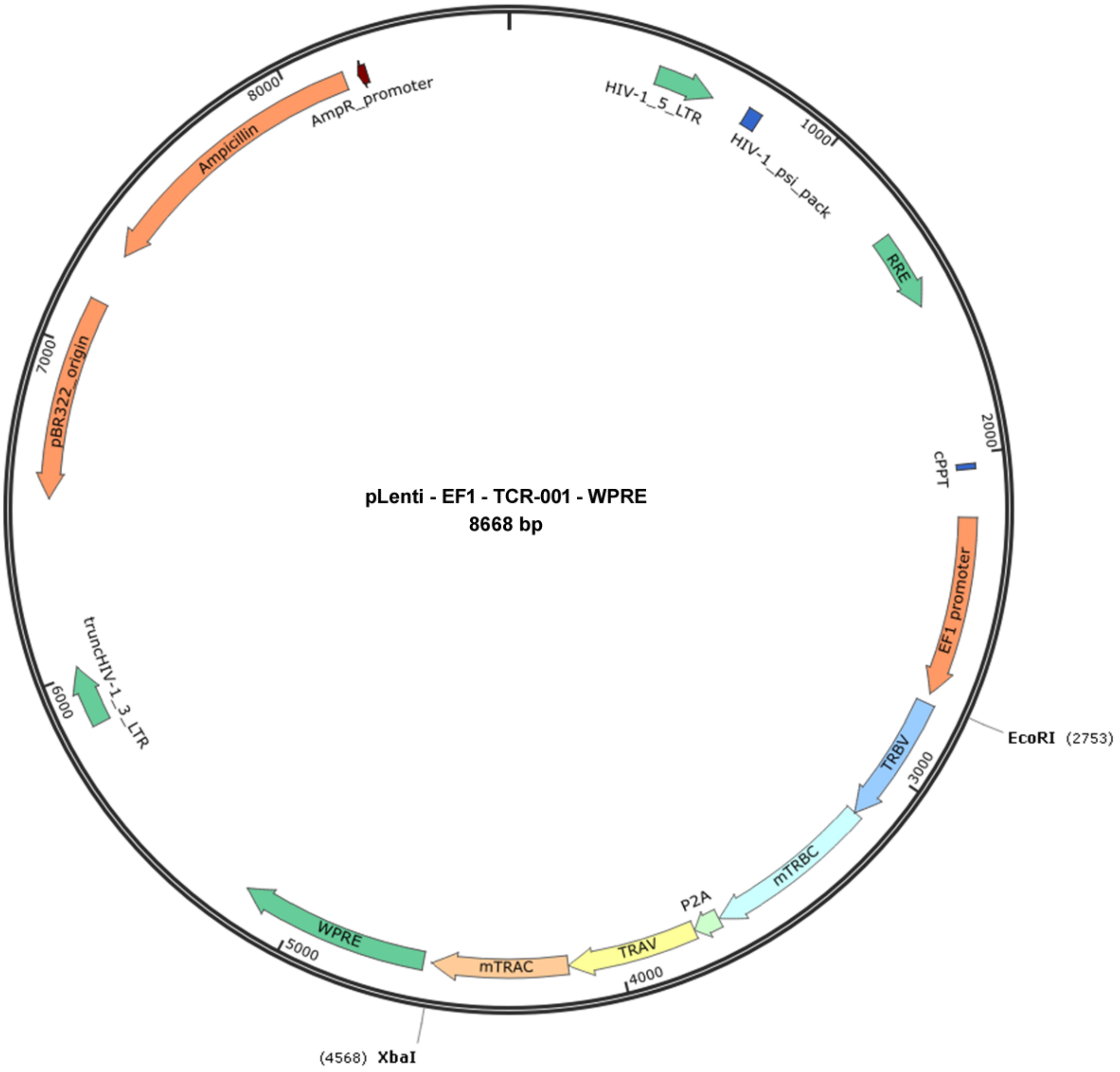

Supplemental Figure 5. Lentiviral vector expressing the fusion of TCR-001 TRBV and TRAV with murine TRBC and TRAC

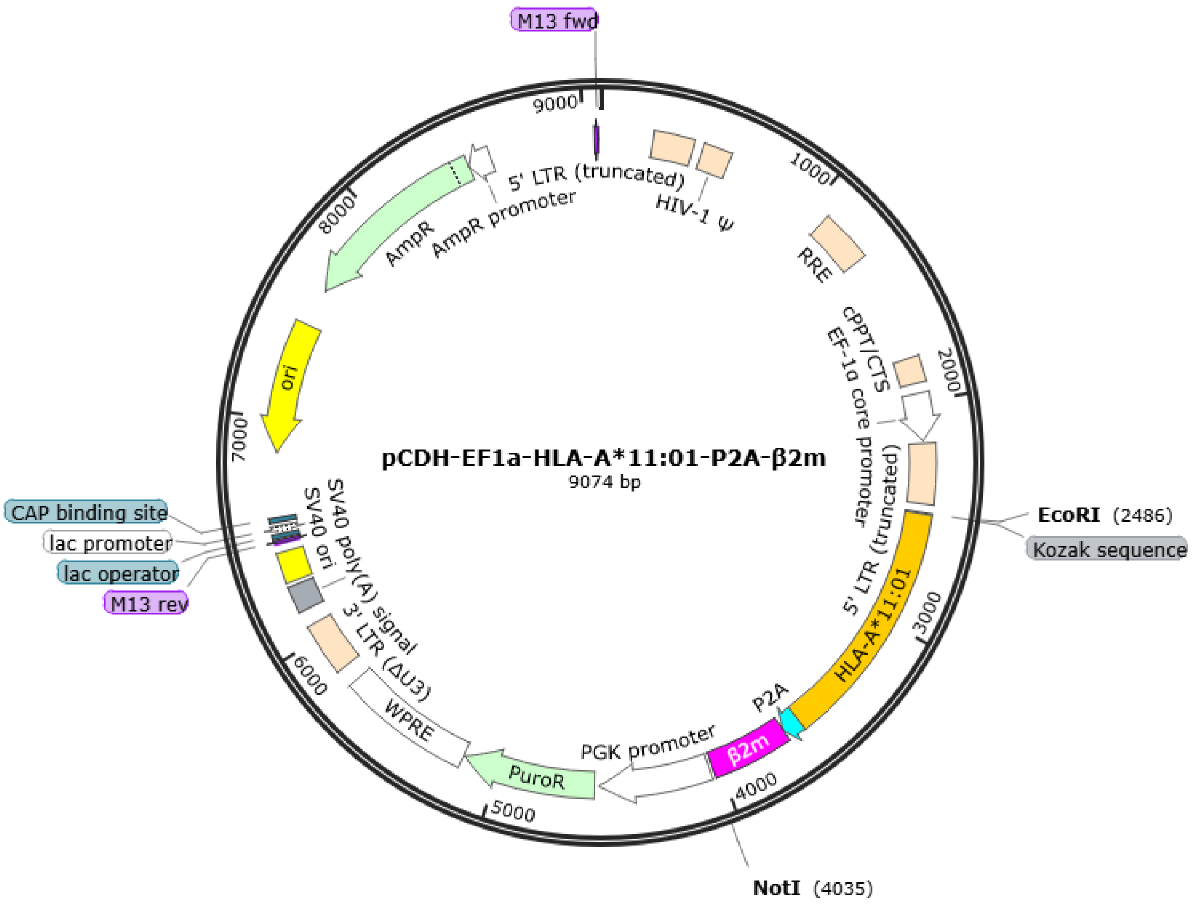

Supplemental Figure 6. The map of lentiviral vector expressing human HLA-A*11:01 and β2m

A

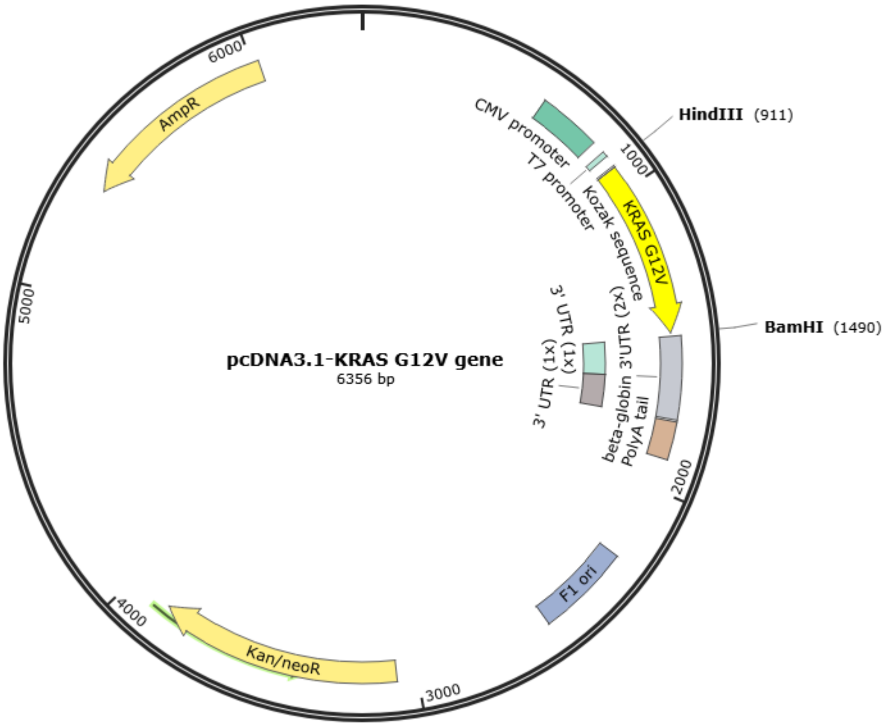

B

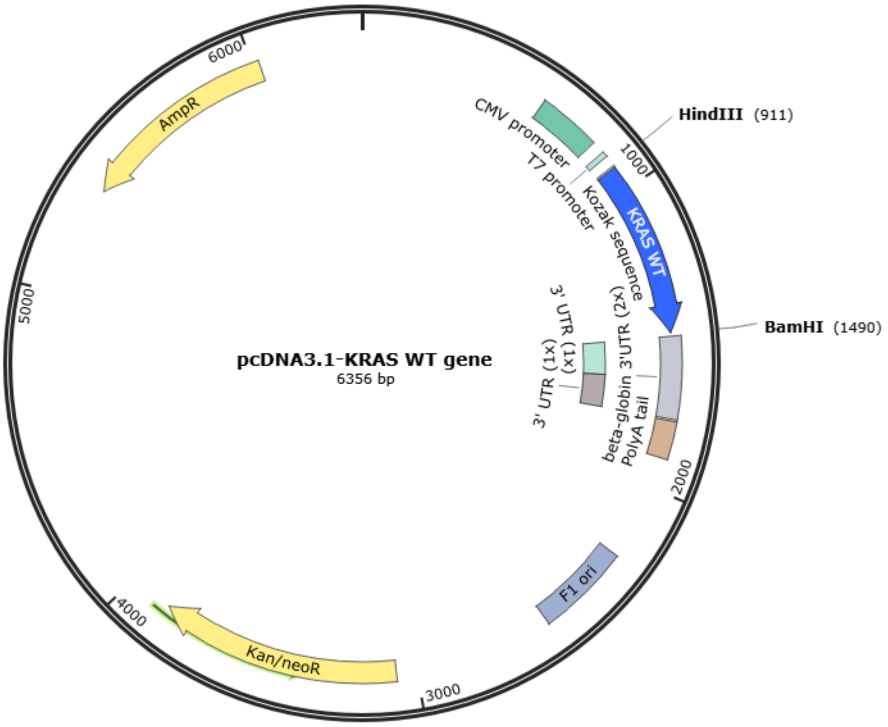

Supplemental Figure 7. The maps of pcDNA3.1 vectors expressing human KRAS G12V full-length gene (A) or human KRAS WT full-length gene (B)

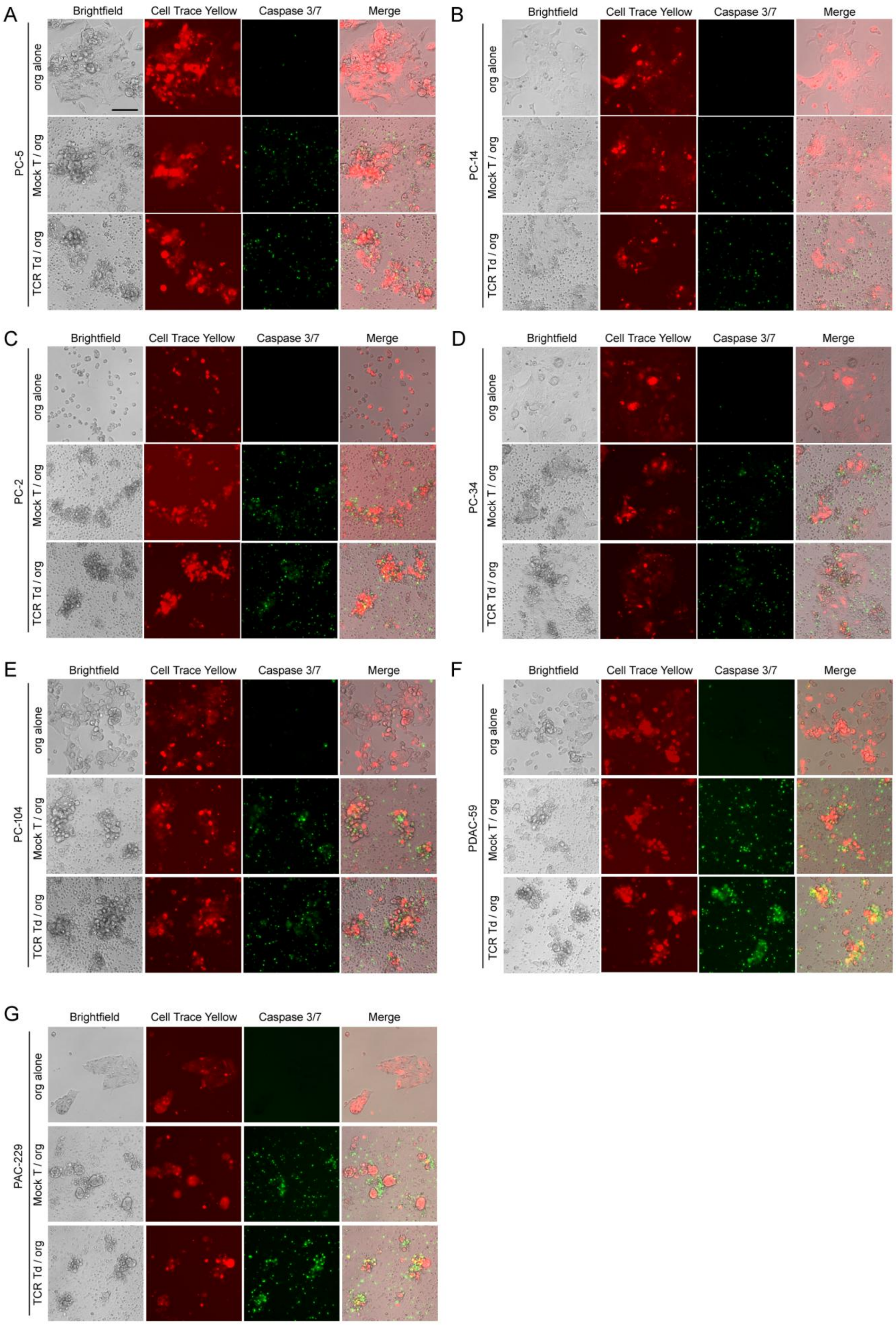

Supplemental Figure 8. TCR-001 transduced T cells specifically

killed only two of five tested human pancreatic cancer organoids

TCR-001 transduced allogeneic T cells or Mock T cells were cocultured with human pancreatic cancer organoid cells naturally expressing KRAS G12V mutations and HLA-A*11:01 on rat tail collagen-coated plates at an effector/target ratio of 10: 1. Human pancreatic cancer organoid cells were labeled with Cell-Trace Yellow before the coculture. After 24 hours of coculture, tumor apoptosis was detected by green-fluorescent Caspase 3/7 probe. Representative microphotographs images with brightfield, Cell-Trace Yellow, Caspase 3/7 green fluorescence and merged figures between Caspase 3/7 green fluorescence and corresponding brightfield and Cell-Trace Yellow were shown. Scale bar, 100 μm.

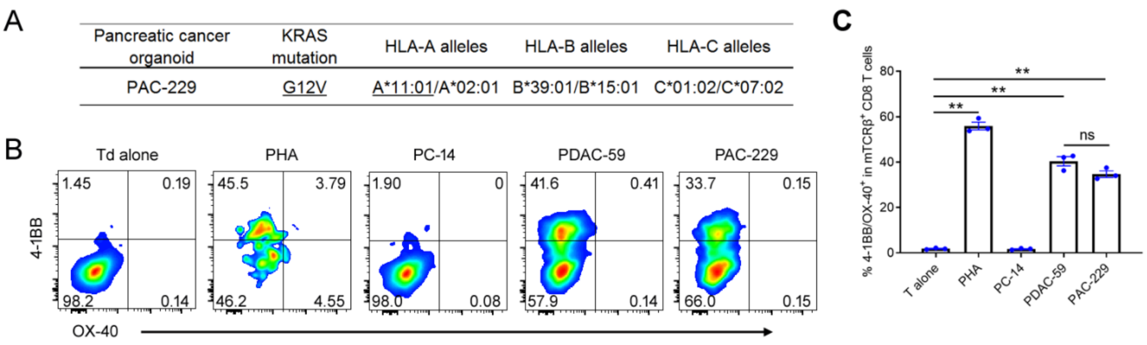

Supplemental Figure 9. TCR-001 transduced T cells specifically recognized human pancreatic cancer organoids

(A) KRAS mutation and HLA profile of human pancreatic cancer organoid PAC-229. (B, C) TCR-001 transduced allogeneic T cells were cocultured overnight with human pancreatic cancer organoid cells naturally expressing KRAS G12 mutations or HLA-A*11:01. 4-1BB/OX-40 upregulation was assayed by flow cytometry. Representative plots (B) and summarized data (C) were shown. The numbers in plots indicated the percentage of different subsets among mTCRβ$^+$ CD8 T cells. Error bars represent SEM of three biological replicates. Dots indicate biological replicates. **, P<0.01; ns, P≥0.05; VS "T alone" group or indicated group. Statistical differences were determined with one-way ANOVA test followed by a post hoc analysis (Tukey's multiple comparison test).

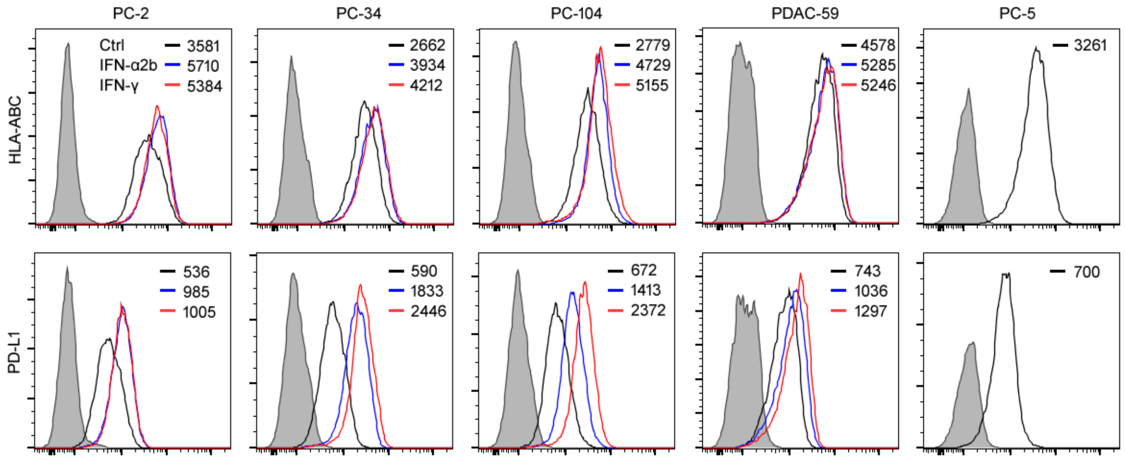

Supplemental Figure 10. HLA-ABC and PD-L1 expression in human pancreatic cancer organoid cells after IFN-α2b or IFN-γ stimulation

Human pancreatic cancer organoid cells were stimulated with 100 ng/mL IFN-α2b or 125 ng/ml IFN-γ for 24 hours. Then single cells were harvested for detecting HLA-ABC and PD-L1 expression by flow cytometry. The numbers in the histograms indicated the geometric mean fluorescence of HLA-ABC or PD-L1 in the test samples. Shaded histograms indicated the test samples without cytokines stimulation and antibody staining. Representative overlay plots from at least three biological replicates were shown.

Supplemental Table 1. Neoantigen prediction of KRAS mutated subtypes by NetMHCpan 4.0

HLA-ABC	KRAS mutation	Peptide	Score	Affinity (nM)	% Rank	Bind Level
HLA-A01:01	G12D	GADGVGKSAL	0.056	27364.68	18.02	
HLA-A02:01	G12D	KLVVVGADGV	0.464	331.62	2.25	
HLA-A02:03	G12D	KLVVVGADGV	0.664	38.13	0.94	WB
HLA-A03:01	G12D	VVVGADGVGK	0.418	541.24	1.28	WB
HLA-A03:01	G12D	VVGADGVGK	0.379	829.57	1.63	WB
HLA-A11:01	G12D	VVGADGVGK	0.522	175.98	0.92	WB
HLA-A11:01	G12D	VVVGADGVGK	0.515	189.62	0.97	WB
HLA-A24:02	G12D	DGVGKSALTI	0.037	33676.06	28.13	
HLA-A30:01	G12D	VVGADGVGK	0.263	2919.05	7.88	
HLA-A68:01	G12D	VVVGADGVGK	0.494	239.22	1.52	WB
HLA-B07:02	G12D	GADGVGKSAL	0.178	7289.44	4.53	
HLA-B08:01	G12D	DGVGKSALTI	0.111	15119.95	18.03	
HLA-B27:05	G12D	YKLVVVGADG	0.076	21980.44	21.17	
HLA-C01:02	G12D	GADGVGKSAL	0.101	16767.12	4.58	
HLA-C03:03	G12D	LVVVGADGV	0.223	4482.67	3.84	
HLA-C03:04	G12D	LVVVGADGV	0.223	4482.67	3.84	
HLA-C04:01	G12D	GADGVGKSAL	0.065	24655.35	7.76	
**HLA-C08:02**	**G12D**	**GADGVGKSAL**	**0.248**	**3425.69**	**0.40**	**SB**
HLA-C08:02	G12D	GADGVGKSA	0.114	14633.72	2.64	
HLA-A01:01	G12V	VVGAVGVGK	0.039	32704.02	31.37	
HLA-A02:01	G12V	KLVVVGAVGV	0.533	156.46	1.42	WB
HLA-A02:03	G12V	KLVVVGAVGV	0.675	33.73	0.84	WB
**HLA-A03:01**	**G12V**	**VVGAVGVGK**	**0.550**	**130.91**	**0.46**	**SB**
HLA-A03:01	G12V	VVVGAVGVGK	0.527	167.67	0.56	WB
**HLA-A11:01**	**G12V**	**VVGAVGVGK**	**0.638**	**50.17**	**0.31**	**SB**
**HLA-A11:01**	**G12V**	**VVVGAVGVGK**	**0.596**	**79.40**	**0.47**	**SB**
HLA-A24:02	G12V	EYKLVVVGAV	0.125	13001.42	6.88	
HLA-A30:01	G12V	VVGAVGVGK	0.422	520.09	2.12	
HLA-A68:01	G12V	VVVGAVGVGK	0.556	121.64	1.03	WB
HLA-A68:01	G12V	VVGAVGVGK	0.478	282.62	1.67	WB
HLA-B07:02	G12V	AVGVGKSAL	0.410	590.34	0.91	WB
HLA-B08:01	G12V	AVGVGKSAL	0.189	6475.64	7.02	
HLA-B27:05	G12V	AVGVGKSAL	0.200	5754.50	5.85	
HLA-C01:02	G12V	AVGVGKSAL	0.152	9700.82	1.81	WB
HLA-C03:03	G12V	GAVGVGKSAL	0.295	2064.71	2.32	
HLA-C03:04	G12V	GAVGVGKSAL	0.295	2064.71	2.32	
HLA-C04:01	G12V	YKLVVVGAV	0.051	28834.33	13.47	
HLA-C08:02	G12V	AVGVGKSAL	0.088	19216.33	4.24	
HLA-A01:01	G12R	VVGARGVGK	0.035	34397.11	37.48	

HLA-A02:01	G12R	KLVVVGARGV	0.445	407.09	2.51	
HLA-A02:03	G12R	KLVVVGARGV	0.662	38.93	0.96	WB
**HLA-A03:01**	**G12R**	**VVGARGVGK**	**0.574**	**100.07**	**0.37**	**SB**
HLA-A03:01	G12R	VVVGARGVGK	0.528	164.67	0.56	WB
HLA-A11:01	G12R	VVGARGVGK	0.565	111.23	0.65	WB
HLA-A11:01	G12R	VVVGARGVGK	0.527	166.74	0.89	WB
HLA-A24:02	G12R	RGVGKSALTI	0.094	18190.78	9.99	
HLA-A30:01	G12R	VVGARGVGK	0.470	311.17	1.39	WB
HLA-A68:01	G12R	VVVGARGVGK	0.502	220.05	1.45	WB
**HLA-B07:02**	**G12R**	**GARGVGKSAL**	**0.544**	**139.68**	**0.37**	**SB**
HLA-B08:01	G12R	GARGVGKSAL	0.213	5000.68	5.44	
HLA-B27:05	G12R	ARGVGKSAL	0.355	1075.59	2.06	
HLA-C01:02	G12R	GARGVGKSAL	0.078	21578.67	7.63	
HLA-C03:03	G12R	LVVVGARGV	0.227	4289.55	3.73	
HLA-C03:04	G12R	LVVVGARGV	0.227	4289.55	3.73	
HLA-C04:01	G12R	ARGVGKSAL	0.050	29154.94	14.00	
HLA-C08:02	G12R	GARGVGKSAL	0.052	28605.94	10.27	
HLA-A01:01	G12C	CGVGKSALTI	0.047	29966.65	23.59	
HLA-A02:01	G12C	KLVVVGACGV	0.508	204.28	1.67	WB
HLA-A02:03	G12C	KLVVVGACGV	0.687	29.51	0.74	WB
HLA-A02:03	G12C	LVVVGACGV	0.571	104.24	1.96	WB
HLA-A03:01	G12C	VVGACGVGK	0.531	159.58	0.54	WB
HLA-A03:01	G12C	VVVGACGVGK	0.509	202.51	0.65	WB
**HLA-A11:01**	**G12C**	**VVGACGVGK**	**0.612**	**66.93**	**0.40**	**SB**
HLA-A11:01	G12C	VVVGACGVGK	0.573	102.06	0.60	WB
HLA-A24:02	G12C	CGVGKSALTI	0.068	23911.59	14.56	
HLA-A30:01	G12C	VVGACGVGK	0.366	953.67	3.36	
HLA-A68:01	G12C	VVVGACGVGK	0.507	207.12	1.40	WB
HLA-B07:02	G12C	GACGVGKSAL	0.140	11012.71	6.53	
HLA-B08:01	G12C	CGVGKSALTI	0.103	16358.38	19.97	
HLA-B27:05	G12C	VVGACGVGK	0.086	19759.02	18.01	
HLA-C01:02	G12C	LVVVGACGV	0.069	23587.80	9.23	
HLA-C03:03	G12C	LVVVGACGV	0.259	3039.43	2.95	
HLA-C03:04	G12C	LVVVGACGV	0.259	3039.43	2.95	
HLA-C04:01	G12C	YKLVVVGAC	0.041	32008.78	20.22	
HLA-C08:02	G12C	LVVVGACGV	0.062	25574.26	7.72	

Score=1-log50k[Affinity]; "SB" indiates strong binding peptide (% Rank<0.5); "WB" indiates weak binding peptide (% Rank<2.0).

Supplemental Table 2. Neoantigen prediction of KRAS mutated subtypes by NetMHC 4.0

HLA-ABC	KRAS mutation	Peptide	Score	Affinity (nM)	% Rank	Bind Level
HLA-A01:01	G12D	LVVVGADGV	0.084	20243.75	10.00	
HLA-A02:01	G12D	KLVVVGADGV	0.426	498.01	3.00	
HLA-A02:03	G12D	KLVVVGADGV	0.618	62.04	1.40	WB
HLA-A03:01	G12D	VVVGADGVGK	0.367	938.80	1.90	WB
HLA-A03:01	G12D	VVGADGVGK	0.347	1172.08	2.50	
HLA-A11:01	G12D	VVGADGVGK	0.454	368.19	1.50	WB
HLA-A11:01	G12D	VVVGADGVGK	0.440	429.98	1.70	WB
HLA-A24:02	G12D	DGVGKSALTI	0.057	27060.25	20.00	
HLA-A30:01	G12D	VVGADGVGK	0.167	8247.88	12.00	
HLA-A68:01	G12D	VVVGADGVGK	0.407	613.21	2.50	
HLA-B07:02	G12D	GADGVGKSAL	0.186	6646.79	5.50	
HLA-B08:01	G12D	GADGVGKSAL	0.140	10987.24	11.00	
HLA-B27:05	G12D	YKLVVVGAD	0.069	23672.69	25.00	
HLA-C03:03	G12D	ADGVGKSAL	0.140	10950.33	5.50	
HLA-C04:01	G12D	ADGVGKSAL	0.091	18774.05	10.00	
HLA-C08:02	G12D	GADGVGKSA	0.109	15390.48	3.50	
HLA-A01:01	G12V	YKLVVVGAV	0.083	20328.70	10.00	
HLA-A02:01	G12V	KLVVVGAVGV	0.473	300.18	2.00	WB
HLA-A02:03	G12V	KLVVVGAVGV	0.618	62.04	1.40	WB
HLA-A03:01	G12V	VVGAVGVGK	0.509	202.62	0.80	WB
HLA-A03:01	G12V	VVVGAVGVGK	0.461	341.99	1.10	WB
**HLA-A11:01**	**G12V**	**VVGAVGVGK**	**0.614**	**65.47**	**0.50**	**SB**
HLA-A11:01	G12V	VVVGAVGVGK	0.545	137.28	0.90	WB
HLA-A24:02	G12V	VGVGKSALTI	0.149	10001.69	6.00	
HLA-A30:01	G12V	VVGAVGVGK	0.290	2174.99	4.00	
HLA-A68:01	G12V	VVVGAVGVGK	0.443	415.56	2.50	
HLA-B07:02	G12V	AVGVGKSAL	0.495	235.33	0.80	WB
HLA-B08:01	G12V	AVGVGKSAL	0.183	6926.82	6.50	
HLA-B27:05	G12V	YKLVVVGAV	0.212	5067.79	6.00	
HLA-C03:03	G12V	AVGVGKSAL	0.418	540.94	1.00	WB
HLA-C04:01	G12V	YKLVVVGAV	0.120	13645.52	5.00	
HLA-C08:02	G12V	YKLVVVGAV	0.068	23906.14	8.50	
HLA-A01:01	G12R	LVVVGARGV	0.067	24275.23	17.00	
HLA-A02:01	G12R	KLVVVGARGV	0.424	506.91	3.00	
HLA-A02:03	G12R	KLVVVGARGV	0.619	61.46	1.40	WB
HLA-A03:01	G12R	VVGARGVGK	0.543	140.14	0.60	WB
HLA-A03:01	G12R	VVVGARGVGK	0.491	245.97	0.90	WB
HLA-A11:01	G12R	VVGARGVGK	0.529	163.14	1.00	WB
HLA-A11:01	G12R	VVVGARGVGK	0.470	308.51	1.40	WB

HLA-A24:02	G12R	RGVGKSALTI	0.188	6545.10	4.50	
HLA-A30:01	G12R	GARGVGKSA	0.532	157.39	0.70	WB
HLA-A68:01	G12R	VVVGARGVGK	0.364	974.78	3.00	
**HLA-B07:02**	**G12R**	**GARGVGKSAL**	**0.583**	**90.73**	**0.40**	**SB**
HLA-B08:01	G12R	GARGVGKSAL	0.198	5857.21	6.00	
HLA-B27:05	G12R	ARGVGKSAL	0.319	1587.84	3.00	
HLA-C03:03	G12R	ARGVGKSAL	0.214	4937.45	3.50	
HLA-C04:01	G12R	YKLVVVGAR	0.132	11922.59	4.00	
HLA-C08:02	G12R	LVVVGARGV	0.041	32190.77	19.00	
HLA-A01:01	G12C	LVVVGACGV	0.093	18286.69	7.50	
HLA-A02:01	G12C	KLVVVGACGV	0.453	373.60	2.50	
HLA-A02:03	G12C	KLVVVGACGV	0.618	62.04	1.40	WB
HLA-A03:01	G12C	VVGACGVGK	0.501	221.43	0.80	WB
HLA-A03:01	G12C	VVVGACGVGK	0.452	375.53	1.10	WB
HLA-A11:01	G12C	VVGACGVGK	0.547	134.97	0.80	WB
HLA-A11:01	G12C	VVVGACGVGK	0.480	278.80	1.30	WB
HLA-A24:02	G12C	CGVGKSALTI	0.119	13828.34	8.50	
HLA-A30:01	G12C	VVGACGVGK	0.238	3790.23	6.00	
HLA-A68:01	G12C	VVVGACGVGK	0.398	676.34	3.00	
HLA-B07:02	G12C	GACGVGKSAL	0.149	9937.83	7.00	
HLA-B08:01	G12C	GACGVGKSAL	0.141	10921.82	11.00	
HLA-B27:05	G12C	YKLVVVGAC	0.119	13784.87	12.00	
HLA-C03:03	G12C	ACGVGKSAL	0.468	316.96	0.80	
HLA-C04:01	G12C	VVGACGVGK	0.126	12734.93	4.50	
HLA-C08:02	G12C	LVVVGACGV	0.042	31785.49	18.00	

Score=1-log50k[Affinity]; "SB" indiates strong binding peptide (% Rank<0.5); "WB" indiates weak binding peptide (% Rank<2.0).

Supplemental Table 3. HLA-II profiles of two patients enrolled.

Patient No.	HLA-DRB1 alleles	HLA-DQA1 alleles	HLA-DQB1 alleles	HLA-DPA1 alleles	HLA-DPB1 alleles
Pt.004	**DRB1*15:01** /DRB1*11:01	**DQA1*01:02** /DQA1*05:05	DQB1*06:02 /DQB1*03:01	DPA1* /DPA1*	**DPB1*02:01** /**DPB1*05:01**
Pt.007	**DRB1*15:01** /DRB1*09:01	**DQA1*01:02** /**DQA1*01:02**	DQB1*06:01 /DQB1*03:03	DPA1*01:03 /DPA1*02:02	**DPB1*02:01** /**DPB1*05:01**

Supplemental Table 4. Clinical characteristics of patients enrolled.

Patient No.	Tumor type	Tumor position	Tumor stage*	Neoadjuvant chemotherapy	PBMC for screening
Pt.001	PDAC	Head of pancreas	IA	No	After surgery
Pt.002	PDAC	Head of pancreas	IB	No	Before surgery
Pt.003	PDAC	Body of pancreas	IIB	No	Before surgery
Pt.004	PDAC	Head of pancreas	IIB	Yes	Before surgery & After surgery
Pt.005	PDAC	Body of pancreas	IA	Yes	Before surgery
Pt.006	PDAC	Body and tail of pancreas	IIB	No	Before surgery
Pt.007	PDAC	Body and tail of pancreas	IIB	No	Before surgery for allo-DC

* AJCC 8th edition

Supplemental Table 5. KRAS mutation and HLA profiles of patients enrolled.

Patient No.	KRAS mutation (Frequency)	HLA-A alleles	HLA-B alleles	HLA-C alleles
Pt.001	**G12V**(31.03%)	**A*11:01**/A*30:01	B*15:01/B*13:02	C*06:02/C*08:01
Pt.002	**G12V**(2.13%)	**A*11:01**/A*24:02	B*39:01/B*38:02	C*07:02/C*07:02
Pt.003	**G12V**(12.17%)	**A*11:01**/A*26:01	B*51:02/B*51:02	C*07:04/C*16:02
Pt.004	**G12V**(2.18%)	**A*11:01**/A*24:02	B*51:01/B*51:01	C*03:03/C*14:02
Pt.005	**G12V**(7.14%)	**A*11:01**/A*02:01	B*13:01/B*51:01	C*03:04/C*14:02
Pt.006	**G12V**(19.2%)	**A*11:01**/A*02:07	B*46:01/B*38:02	C*07:02/C*01:02
Pt.007	**G12D**(6.85%)	**A*11:01**/A*02:07	B*55:02/B*13:01	C*01:02/C*03:04

Supplemental Table 6. Key Resources in this study

REAGENT or RESOURCE	SOURCE	IDENTIFIER	CLONE TYPE (For Antibodies)
**Antibodies**			
Anti-human CD3 Pacific blue	Biolegend	Cat#317314	Clone: OKT3
Anti-human CD8 PE/Cy7	Biolegend	Cat#344712	Clone: SK1
Anti-human CD4 APC/Fire™ 750	Biolegend	Cat#344638	Clone: SK3
Anti-human CD137 APC	Biolegend	Cat#309810	Clone: 4B4-1
Anti-human CD134 FITC	Biolegend	Cat#350006	Clone: Ber-ACT35 (ACT35)
Anti-human CD3 (OKT3)	Biolegend	Cat#317326	Clone: OKT3
Anti-human CD45RO FITC	Biolegend	Cat#304242	Clone: UCHL1
Anti-human CD62L APC	Biolegend	Cat#304810	Clone: DREG-56
Anti-human Fas Percp Cy5.5	Biolegend	Cat#305630	Clone: DX2
Anti-human PD1 FITC	Biolegend	Cat#329904	Clone: EH12.2H7
Anti-human CD-107a PE	Biolegend	Cat#328608	Clone: H4A3
Anti-human IFN-γ APC	Biolegend	Cat#506510	Clone: B27
Anti-human TNF-α Percp Cy5.5	Biolegend	Cat#502926	Clone: MAb11
Anti-mouse TCR-β FITC	Biolegend	Cat#109206	Clone: H57-597
Anti-mouse TCR-β PE	Biolegend	Cat#109208	Clone: H57-597
Ultra-LEAF™ Purified anti-human HLA-A,B,C Antibody	Biolegend	Cat#311428	Clone: W6/32
Ultra-LEAF™ Purified anti-human	Biolegend	Cat#307648	Clone: L243

HLA-DR antibody			
Anti-human HLA-DP monomorphic purified	Leinco Technologies	Cat#H127	Clone: B7/21
Purified anti-human HLA-DQ antibody	Biolegend	Cat#361502	Clone: Tü169
Anti-human PD-1 humanized monoclonal antibody	BeiGene	Cat#BGB-A317	
**Chemicals, peptides, and recombinant proteins**			
T Cell TransAct™, human	Miltenyi Biotec	130-111-160	2×2 mL
Monensin solution (1,000X)	Biolegend	Cat#420701	
Brefeldin A solution (1,000X)	Biolegend	Cat#420601	
LPS	Sigma-Aldrich	Cat#L2654-1MG	
R848	Sigma-Aldrich	Cat#SML0196-10MG	
Custom peptides (HPLC)	Sangon	Custom order	
Recombinant Human GM-CSF	Novoprotein	Cat#GMP-CC79	
Recombinant Human IL-4	Novoprotein	Cat#GMP-CD03	
Recombinant Human IL-7	Novoprotein	Cat#GMP-CD47	
Recombinant Human IL-2	Novoprotein	Cat#GMP-CD66	
Recombinant Human IFN-α2b	Novoprotein	Cat#C005	
Recombinant Human IFN-γ	Novoprotein	Cat#GMP-CI57	
Matrigel	Corning	Cat#356231	
Matrigel	Corning	Cat#354234	
CellTrace™ Yellow	Invitrogen	Cat#C34573	
CellEvent™ Caspase-3/7 Green	Invitrogen	Cat#C10723	

Detection Reagent			
**Commercial assay kits**			
Human INF-γ ELISPOT assay kit	Dakewe	Cat#2110005	
